# Similar Associations of Tooth Microwear and Morphology Indicate Similar Diet across Marsupial and Placental Mammals

**DOI:** 10.1371/journal.pone.0102789

**Published:** 2014-08-06

**Authors:** Hilary B. Christensen

**Affiliations:** 1 The University of Chicago, Department of Geophysical Sciences, Chicago, Illinois, United States of America; 2 Bates College, Geology Department, Lewiston, Maine, United States of America; University of Oxford, United Kingdom

## Abstract

Low-magnification microwear techniques have been used effectively to infer diets within many unrelated mammalian orders, but the extent to which patterns are comparable among such different groups, including long extinct mammal lineages, is unknown. Microwear patterns between ecologically equivalent placental and marsupial mammals are found to be statistically indistinguishable, indicating that microwear can be used to infer diet across the mammals. Microwear data were compared to body size and molar shearing crest length in order to develop a system to distinguish the diet of mammals. Insectivores and carnivores were difficult to distinguish from herbivores using microwear alone, but combining microwear data with body size estimates and tooth morphology provides robust dietary inferences. This approach is a powerful tool for dietary assessment of fossils from extinct lineages and from museum specimens of living species where field study would be difficult owing to the animal’s behavior, habitat, or conservation status.

## Introduction

Mammalian teeth, being both obviously relevant to feeding and well preserved in the fossil record, have been the focus of dietary reconstructions for generations [1] and the emerging field of dental ecology [2] takes advantage of the varied tools teeth provide for dietary analyses. One of the most frequently used techniques in recent years has been the quantification of microwear damage incurred by tooth enamel surfaces during mastication. Although the mechanisms involved in tooth wear are complex, microwear is thought to be correlated to the abrasiveness and physical properties of an animal’s diet [3]–[8]. Wear patterns are constantly overwritten and probably reflect dietary habits over a relatively short period of time; this has been estimated at days to weeks for features observable by SEM, depending upon the region of the tooth observed and the abrasiveness of the diet [9]. As such, microwear has been used to track dietary changes in mammals over seasonal as well as paleontological time scales. Such analyses have generally been restricted to comparisons within a single clade, including primates [2], [10]–[16], xenarthrans [17], mammoths [18], [19], carnivores [20]–[23], ungulates [24]–[29], rodents [30]–[33], fish [34], bats [23], [35] and macropod marsupials [36]. In such studies within lineages, animals with unknown diets can be compared to closely related taxa whose diets are better understood (via gut contents or controlled feeding experiments, e.g. [7], [37], [38]). The extent to which microwear comparisons might be suitable between more distantly related mammalian lineages, including fossils without close living relatives, has not been assessed.

Microwear damage to teeth is related to the incidental ingestion of hard particles (e.g. phytoliths, bone fragments, and especially exogenous silica) during mastication [22], [39]–[43]. In mammals, different microwear patterns occur because different types of chewing strokes are associated with different diets. Mammals emphasizing compressive chewing, most effective for the processing of hard, brittle foods (nuts, seeds, bone) via crack propagation have proportionally more pits comprising their microwear patterns than mammals emphasizing the grinding or shearing associated with tough or ductile foods (e.g. leaves, grass, flesh), which tend to have microwear signatures dominated by striations rather than pits (e.g. [44]–[51]). Early microwear studies took advantage of the different proportions of striations (shearing related damage) vs. pitting (crushing related damage) on the teeth of mammals with different diets, quantifying these features from SEM images (e.g. [52]–[54]). Generally, a higher proportion of scratches relative to pits is interpreted as being reflective of consumption of tough foods, with the reverse reflecting brittle food consumption [55]–[57]. The time and expense involved in these analyses led to the development of low-magnification light microscopy techniques (LDM) [28], which follow similar methods to SEM but allow quick and low-cost analysis; damage feature frequencies are counted in fields of view ranging from 0.01 mm^2^ [30] to 0.4 mm^2^ [28] depending upon the size of the animal.

Both SEM and LDM techniques rely on observer measurements and therefore direct comparison of results between different methodologies and users can be difficult. Inter-observer error rates have been estimated at 5–6% in users counting from an SEM image [58], [59]. LDM estimates put absolute feature frequency error rates at about 9%, although subsequent statistical testing showed highly significant differences between the assigned dietary categories even when inter-observer error is included in the analysis [60]. Nevertheless, concerns about repeatability between observers led to the development of microwear techniques focusing on the measurement of features from a photograph [61], [62]. Achieving uniformly adequate image quality can be limiting [63], [64], however, particularly when a wide diversity of tooth morphologies and sizes are involved, often requiring fine adjustments to focus across a field of view. Error rates associated with analyzing LDM photos range from 45% in inexperienced users to 8% in experienced individuals [64].

3D dental microwear texture analysis (DMTA) techniques have been developed to alleviate inter-observer bias by automating the recognition of microwear features [21], [65]–[67], and have been shown to discriminate diets successfully in a variety of mammalian lineages. DMTA employs scale-sensitive fractal analysis (SSFA) or International Organization for Standardization three-dimensional surface texture parameters (ISO 25178-2) derived from surface elevation data. Dietary discrimination is based on multiple parameters related to the topography of the surface, as opposed to the two variables generally measured in SEM and LDM analyses (scratches and pits, although distinguishing of different size classes of pits and scratches may amplify the number of variables). DMTA has shown the potential to have more discriminatory power than 2D methods when assigning diet, especially within a single lineage [35], [68], and even to reveal inter-individual dietary differences within a single species [69]. However, the much smaller tooth area sampled with this technique can lead to variability between analyzed patches (e.g. Phase I and Phase II wear facets in primates [70]). While useful if intra-tooth variation is the object of study, this also necessitates great care and uniformity in choosing regions of the tooth to analyze if dietary comparisons between taxa are required, and perhaps limits comparisons between more distantly related lineages with very different tooth architectures.

Different microwear methodologies have different strengths and weaknesses and all have value depending upon the scientific questions being addressed. The goal of this study is to develop a suite of techniques useful for the assignment of very broad dietary categories across the mammals, irrespective of the animal’s lineage. LDM microscopy (direct counting through the lens) was chosen as the microwear methodology most suitable for this study due to the high throughput of samples required and the wide availability of LDM to researchers in a variety of fields. Although training in the method is required, no equipment is needed other than a stereomicroscope. LDM studies analyze a larger proportion of the total tooth surface than do DMTA techniques, giving more representative coverage of the tooth and making comparisons across a wide variety of mammalian lineages more feasible. While recognizing that this method allows differentiation of coarser dietary bins than does DMTA, LDM has been shown in numerous studies to discriminate reliably between broad herbivorous dietary categories (i.e. frugivore, grazer, browser) [60] as well as bone- vs. flesh-consuming carnivores [68].

A study using LDM to distinguish a broader range of dietary categories (i.e. grazer, browser, carnivore, insectivore) in the same analysis, however, has not yet been undertaken and complications arise when considering different types of foods that possess similar physical properties. For example, flesh-consuming carnivores and browsing ungulates have been found in some cases to have overlapping microwear patterns [71]: both eat tough or ductile foods that require a shearing motion for breakdown and that incorporate relatively little grit. Complications stemming from the relatively coarse discriminatory power of LDM microwear analysis, such as the difficulty of identifying specific food items with similar fracture modes, demonstrate that additional lines of evidence are required when developing a proxy for the determination of diet in a mammal with a truly unknown biology.

The two main strategies for oral processing (shearing and crushing) are also reflected in tooth shape [51], [72]–[78]. Mammals with diets requiring vertical crushing for breakdown have molars characterized by low, rounded cusps and few enamel ridges. A diet composed of tough foods, by contrast, requires transverse shearing movements and fosters the evolution of molars with ridges of enamel (shearing crests) that come into contact between upper and lower teeth during mastication to serve as cutting surfaces [73]–[78]. Mammals emphasize shearing vs. crushing surfaces on their cheek teeth (relative to other members of the same clade) depending on diet, shown in a number of studies of living and fossil mammals [78]–[84]. Even foods with similar physical properties can in some cases be associated with distinct tooth morphologies. Both leaf and meat eaters require shearing forces to process their food, however reduction of ingested foods to small particle size is of particular importance for herbivores [85], [86] due to the difficulties involved in breaking down plant cell walls. Thus, herbivore and carnivore teeth are distinct, with more and/or longer shearing crests typically present in herbivores relative to carnivores of similar sizes. Since tooth shape reflects selection for efficient processing of a particular type of diet over evolutionary timescales versus the microwear damage directly caused by foods recently consumed by the individual, tooth shape and microwear can provide independent sources of complementary information. This increases the discriminatory power of dietary analysis and can also reveal cases in which microwear and morphological data are seemingly non-correlated, providing additional information about an animal’s ecology (e.g. [87]). An adaptation of a combined microwear/morphological technique to comparison of dietary information across different mammalian clades, however, has not yet been undertaken.

Body size–as can be estimated from molar tooth size [88], [89]– also places important constraints on diet. Whereas carnivores, insectivores, and hard-object feeders (fruits, nuts, seeds) can rely on “auto-enzymatic” digestion, molecular breakdown by enzymes produced by the animal itself, herbivores eating high fiber plant matter require bacterial symbionts to break down plant cellulose (e.g. [90]–[93]). Thus, the lower quality of high-fiber plant matter (relative to meat, fruit, or seeds) requires either a long residence time in the gut to increase the digestive yield or a high throughput at low yield. Both of these alternatives would be limited by small body size, with the minimum estimated to be at about 500 g for extant mammals subsisting entirely on leaves (e.g. [94], [95]). Below this threshold, increasing degrees of omnivory are required. Thus, body mass can constrain ecology and also be a useful indicator of an animal’s diet in addition to the tooth characteristics discussed above.

The goal of this study is to develop a widely applicable and widely available analytical protocol for the assignment of diet in mammals by augmenting LDM microwear with additional dietary metrics. Like microwear, neither body mass nor tooth morphology is diagnostic of diet when standing alone, but each can narrow the pool of potential dietary guilds to which a mammal might belong. Analytical techniques individually evaluating LDM microwear, body mass, and tooth morphology have been used effectively in numerous dietary studies, however these analyses are typically limited to comparisons within a single extant mammalian clade, and rarely used in combination with one another. It is unclear how consistent microwear patterns and shearing crest lengths should be in more distantly related lineages with similar diets but different jaw mechanics, and whether microwear and morphological analyses should be trusted in extinct lineages without close extant relatives. To fill this gap in current knowledge, an analytical protocol is developed for the assignment of broad dietary categories (grazing, browsing, hard-object feeding, insectivory, bone-dominated carnivory, flesh-dominated carnivory) in mammals using the combined information available from body mass, microwear, and tooth morphology. The extent to which these variables may be used as predictors of ecological niche in phylogenetically divergent lineages is tested by taking advantage of the frequent convergence in diet between extant placental (eutherian) and marsupial (metatherian) mammals despite at least 100 million years of separate evolution [96].

## Methods

### Sampling Strategy

All of the animal species chosen for analysis were dietary specialists: browsers (leaves of trees and shrubs), grazers (grass and forbs), hard-object feeders (fruits, nuts, seeds), insectivores (cuticle-bearing), bone carnivores, or flesh carnivores. Dietary information was taken from the literature [97], based on observations in the wild; all specimens were wild-shot. Specialized feeders on a single food type minimize the number of unknown variables affecting the observed tooth morphology and microwear.

Casts of tooth crowns were made on-site at the Field Museum of Natural History (Chicago, IL) and the American Museum of Natural History (New York, NY). Following standard techniques [60], 3M ESPE Express vinylpolysiloxane molding compound (light body, regular set) and clear Buehler Epo-Kwik epoxy resin casting material were used to replicate tooth crowns. The second molar is generally preferred for morphological and microwear analyses in most animals due to its intermediate degree of wear (less than M1, more than M3). However, the molars are often reduced or absent in carnivores where instead the carnassials (equivalent to the upper P4 in extant carnivorans) are modified for food processing. Thus, the upper left second molar was sampled for all taxa except carnivores, for which the upper left carnassial was substituted, following literature convention [20], [43], [98]. In all, 153 extant species belonging to 9 orders were analyzed, including 111 placental and 42 marsupial species (see Table S1 in Supporting Information S1 for complete list). A maximum of eight individuals were sampled from each species, resulting in an overall sample of 247 eutherian and 146 metatherian teeth.

### Tooth measurements

**Figure 1:**
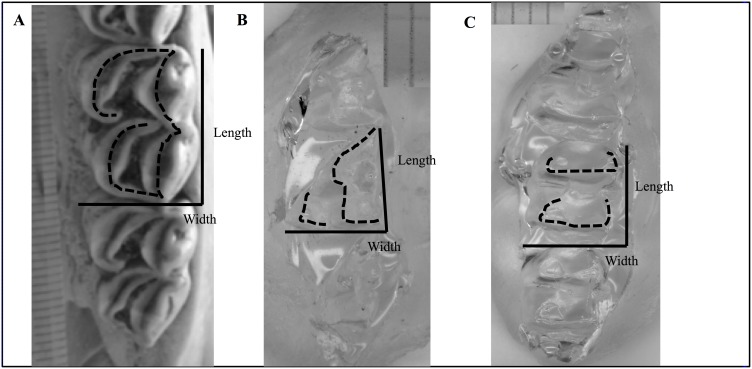
Variables measured for morphometric analysis. Length, width, and total shearing crest length were measured on upper second molars. Morphology varies with phylogeny; examples shown here are A) *Rangifer tarandus* (tooth *in situ)*, B) *Perameles nasuta* (tooth cast), and C) *Dorcopsis hageni* (tooth cast). Tic marks are 1 mm.

Morphometric analysis followed modifications of previous techniques [83], [99]. Tooth casts were imaged with a flatbed scanner in occlusal view, except in the case of teeth too large to be fully cast, which were instead photographed *in situ* in occlusal view. Tooth length and width were measured in ImageJ (available at http://rsbweb.nih.gov/ij/), as was the total length of the shearing crests (Figure 1). Analysis of the three-dimensional crests from a two-dimensional image will underestimate their total length, but two dimensions are nonetheless adequate to distinguish ecological guilds, as demonstrated below. The measured shearing crest length was then divided by the square root of the molar crown area (length×width) to calculate the Shearing Crest Score (SCS) (see Table S2 in Supporting Information S1 for complete taxon list). This new variable was developed because the more established Shearing Quotient (crest length divided by molar length) [99], [100] was developed for use only with lower teeth. Because opposing molars are generally the same size, taking the square root of the crown area provides a linear unit of measure that is independent of shape. As one of the main goals of this study is to develop a proxy for use in the mammalian fossil record, which is composed largely of isolated teeth and in which sample size is usually an issue, a technique that would be applicable to both upper and lower molars is preferable. Average species body masses were taken from the literature [101]; the published mass is the average between males and females in cases with noticeable dimorphism.

**Figure 2:**
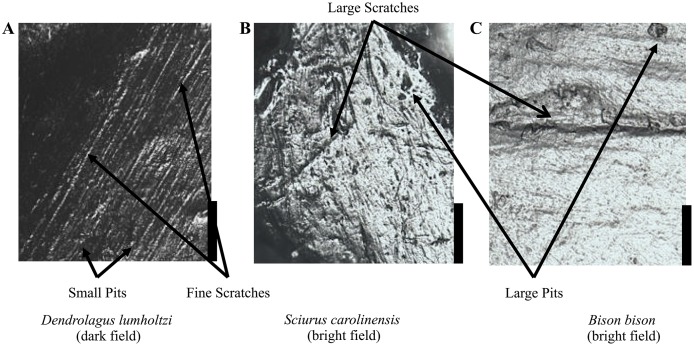
Microwear features tallied during analysis. Large features show up dark on a light field (A); small scratches and small pits show up light on a dark field. (B and C) Dark field versus bright field illumination is achieved by adjusting the light source angle (see also Figure S1 in Supporting Information S1). Scale bars are 0.5 mm.

Dental microwear analysis was carried out with low magnification light microscopy, following previously developed protocols [28], [60] modified to accommodate a wider range of tooth sizes. Features were counted in a 0.04 mm^2^ reticle grid at 70x (a 3 mm diameter field of view) on an Olympus SZX16 stereomicroscope. Microwear features tallied included number of small pits (*P_s_*), number of large pits (*P_l_*), number of fine scratches (*S_f_*), and number of coarse scratches (*S_c_*) (Figure 2). The total number of scratches (*S_t_*) and total number of pits (*P_t_*) were calculated by adding the two size classes together. Adjustment of the unidirectional light source provided a qualitative measure of feature depth: small features appear light on a dark background, while large features appear dark on a light background (see Section 3 and Figure S1 in Supporting Information S1 for details or [60] for a more detailed review). Each measurement consisted of light and dark background counts on the same area done back-to-back in order to ensure that identical areas were counted and to prevent double-counting features.

Most molar measurements were made on the M2 protocone, a shared morphological character among all mammals studied and a different area than is generally analyzed in LDM studies. Because of the great variety of tooth morphologies and chewing mechanisms encompassed by the animals in this study, choosing a single Phase I or Phase II facet for analysis (as is commonly done in primates [70]) was impossible. Where permitted by tooth size, up to four measurements were taken on each tooth (within a single wear facet) in order to calculate intra-tooth variation. All measurements included were made by the author over a period of three months, eliminating inter-observer bias and minimizing intra-observer variability over time.

### Data analysis

Statistical analyses were carried out in SPSS v. 22.0.0 (SPSS Inc., Chicago, US). All count data were log-transformed to achieve homoscedasticity (evaluated with residual linear regression plots) and normality (evaluated with Shapiro-Wilk tests) prior to analysis. An independent-samples t-test (comparing two groups) or a univariate ANOVA (more than two groups) was coupled with a post-hoc least significant difference (LSD) test to determine the significance of individual variable pairings. To test for the combined influence of multiple variables, nested ANOVA (two variables) and Linear Discriminant Analysis (LDA) (more than two variables) were then performed using variables found to have discriminating power between dietary guilds. Post-hoc leave-one-out classification results were employed with LDA to evaluate the degree to which the resulting clusters could be distinguished. The significance of LDA was assessed using Wilks’ Lambda. Statistical tests were performed on both the global data set, which includes all sampled teeth, as well as a set comprised only of species with three or more individuals represented (referred to hereafter as the “limited data set”). The limited data set serves as a check on microwear variability sourcing from species represented by limited numbers of individuals, and yielded the same results as the global data set for all statistical tests performed (for details, see Section 4 (Table S3 and S4 in Supporting Information S1)); thus, the results presented below represent the more conservative global data set.

## Results

**Figure 3:**
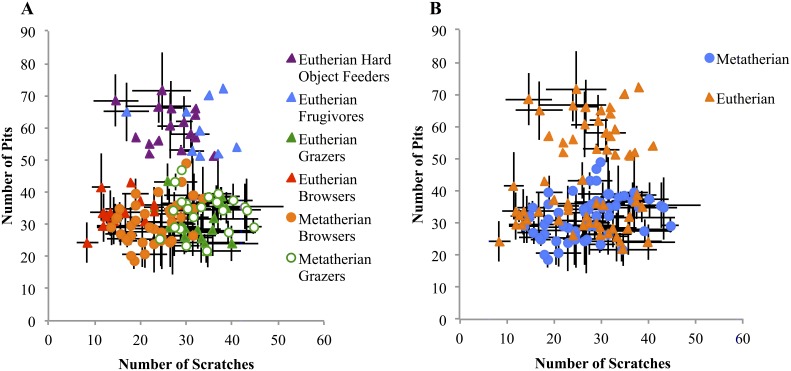
Microwear patterns of marsupial and placental herbivores plotted according to feeding guild (A) and phylogeny (B). Note, the partial separation between marsupials and placentals in B is only based upon the absence of obligate hard object feeders (and, thus, of elevated pit counts) among marsupials in the data set. The three dietary groupings in A are significant (p<0.01). Marsupial and placental herbivores of comparable guilds are statistically indistinguishable (p = 0.84).

Plotting the total pits vs. total scratches for three herbivorous guilds (browsers, grazers, and hard-object feeders) resulted in the “dietary triangle” typically observed in LDM analyses [60] (Figure 3). Grazers form the lower right corner, with many scratches but few pits; leaf browsers have a similar, slightly more variable number of pits and fewer scratches. The apex is formed by the hard-object feeders, which have many more pits than either grazers or browsers. These three groups, based on total pit and scratch counts, are significant (Nested ANOVA, total pit and scratch counts as dependent data, type of measurement and diet as nested factors, Grazers:browsers p = 0.022, browsers:hard-object p<0.0001, grazers:hard-object p = 0.004; limited data set, Grazers:browsers p<0.0001, browsers:hard-object p<0.0001, grazers:hard-object p<0.0001). Obligate hard-object feeding or frugivory is extremely uncommon among Australidelphids, so this dietary habit could not be directly compared. Eutherian hard-object feeders were removed from analyses testing for differences based on phylogeny. A nested ANOVA (total pit and scratch counts dependent, type of measurement and lineage as nested factors) showed the metatherian and eutherian groups to be not significantly different (p = 0.84).

**Figure 4:**
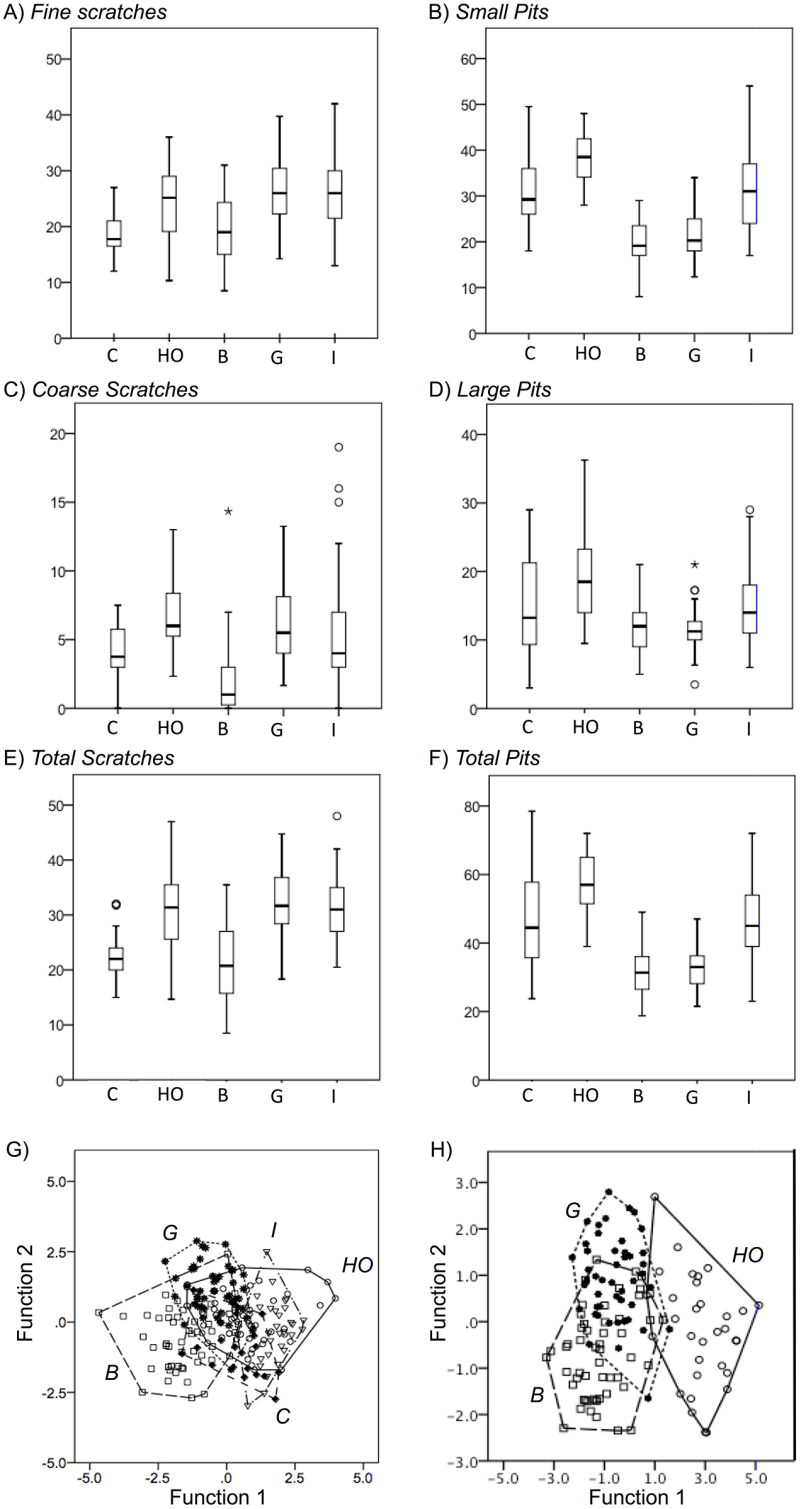
The relative frequencies of microwear variables within feeding guilds. A) Fine scratches (*S_f_*), B) Small Pits (*P_s_*), C) Coarse Scratches (*S_c_*) D) Large pits (*P_l_*), E) Total scratches, F) Total pits. Box plots show the median (center line), interquartile range (boxes), 1.5 times the interquartile range (whiskers), and outlier points. Discriminant analysis using the four independent variables (*S_f_, S_c_, P_s_, P_l_*) as dependents is depicted: G) all five feeding guilds included. Functions 1 and 2 are plotted and account for 71.8% and 24.6% of the variance, respectively. Wilks’ Lambda = 0.252, Chi-Square = 250.51, p<0.001. The discriminant function coefficients are *S_f_*_0.039/0.578_, *S_c_*_0.499/0.688_, *P_s_*_0.845/−0.416_, *P_l_*_0.071/−0.166_. F) Herbivores groups only (grazers, browsers, hard-object feeders). Functions 1 and 2 are plotted and account for 75.1% and 24.9% of the variance, respectively; Wilks’ Lambda = 0.194, Chi-Square = 192.80, p<0.001. The discriminant function coefficients are *S_f_*_0.005/0.379_, *S_c_*_0.324/0.844_, *P_s_*_0.822/−0.330_, *P_l_*_0.270/−0.276_. HO = Hard-Object Feeders, B = Browsers, G = Grazers, I = Insectivores, C = Carnivores. Hulls surround each dietary group.

**Figure 5:**
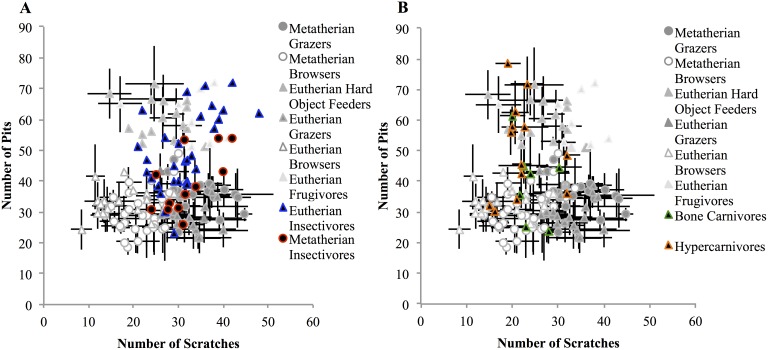
Microwear results of insectivores (A) and carnivores (B) plotted with herbivorous taxa. Marsupial and placental insectivore groups are not significantly different (p = 0.65), nor are hypercarnivores (flesh eaters) and bone carnivores (chew and consume bone) (see Table S5 in Supporting Information S1 for details). Regardless of feeding preferences, both flesh and bone specialists consume both types of food, depending on availability, the season, the animal’s status in social groups, and other factors, resulting in a lack of further differentiation.

Adding the carnivore and insectivore guilds reveals overlap in feature frequency between animals with plant- and animal-based diets. Flesh- and bone-consuming carnivores were treated as a single group because no statistical difference could be found between the two using the LDM methods employed (see Table S5 in Supporting Information S1). Figure 4 shows box plots of all variables tallied during microwear analysis. Results of univariate ANOVA and post-hoc LSD tests for each variable are shown in Table 1. Carnivores and browsers generally have a lower frequency of scratches, *S_f_, S_c_*, and *S_t_*. Pit frequencies, on the other hand, are generally higher in carnivores, hard-object feeders, and insectivores. The discriminatory power of LDM microwear alone as a diagnostic dietary feature begins to fail when considering animals feeding on items that can have similar physical properties, such as carnivores vs. browsers (LSD Post-Hoc, *S_c_*_C-B_ *= *0.868, *S_t_*_C-B_ = 0.133, *P_l_*_C-B_ = 0.145), or carnivores vs. insectivores (LSD Post-Hoc, *S_c_*_C-I_ *= *0.285, *P_s_*_C-I_ = 0.672, *P_l_*_C-F_ = 0.691, *P_t_*_C-F_ = 0.754). Insectivory, carnivory, and hard-object feeding are particularly problematic because of the variation involved (Figure 5A, B); all rely on patchily distributed resources, and animals belonging to any of these guilds may encounter a variety of food items. LDA of the four microwear parameters (*S_f_, S_c_, P_s_*, and *P_l_*) reveals separation between the three herbivorous guilds in dietary space (Figure 4H). Canonical discriminant function 1 (75.1% of variance) is strongly correlated with small pits (*P_s = _*0.892), while function 2 (24.9% of variance) is strongly correlated with coarse scratches (*S_c_* = 0.798). Hard-object feeders, which have a higher proportion of pits to scratches, have positive function 1 values while browsers and grazers tend to have more negative values. Grazers generally to have a higher proportion of coarse to fine scratches ( = positive values) than browsers do (negative values), although there is some overlap between these two guilds on function 2. The “dietary space” defined by this analysis is therefore hallmarked by increasing numbers of small pits on function 1 and increasing numbers of coarse scratches on function 2. This LDA test of the three herbivorous guilds resulted in defined clusters and a post-hoc leave-one-out correct assignment rate of 82.8% (limited data set 84.2%), however the same test including all five guilds had a correct assignment rate of only 57.2% (limited data set 57.1%) and substantial overlap between insectivores, carnivores and hard object feeding herbivores (Figure 4G). Because of this, reliable dietary assignment for an animal not known for sure to be an herbivore requires additional lines of evidence, as outlined below.

**Table 1:** Microwear differences between feeding guilds.

					LSD Post-Hoc Results: p-values									
	Variable	*F*	*d.f.*	*p*	C-HO	C-B	C-G	C-I	HO-B	HO-G	HO-I	B-G	B-I	G-I
Microwear:Scratches	*S_f_*	12.85	4	<0.001	**0.011**	0.868	**<0.001**	**<0.001**	**0.003**	0.061	0.081	**<0.001**	**<0.001**	0.869
	*S_c_*	27.26	4	<0.001	**0.005**	**<0.001**	**0.019**	0.285	**<0.001**	0.425	**0.023**	**<0.001**	**<0.001**	0.099
	*S_t_*	28.40	4	<0.001	**<0.001**	0.133	**<0.001**	**<0.001**	**<0.001**	0.185	0.615	**<0.001**	**<0.001**	0.353
Microwear:Pits	*P_s_*	52.06	4	<0.001	**0.001**	**<0.001**	**<0.001**	0.672	**<0.001**	**<0.001**	**0.001**	0.243	**<0.001**	**<0.001**
	*P_l_*	10.60	4	<0.001	**0.002**	0.145	0.07	0.691	**<0.001**	**<0.001**	**0.001**	0.608	**0.016**	**0.005**
	*P_t_*	49.41	4	<0.001	**<0.001**	**<0.001**	**<0.001**	0.754	**<0.001**	**<0.001**	**<0.001**	0.568	**<0.001**	**<0.001**
Body Mass	*BM*	49.35	4	<0.001	**<0.001**	0.156	0.662	**<0.001**	**<0.001**	**<0.001**	**0.037**	**0.005**	**<0.001**	**<0.001**
Shearing CrestScore	*SCS*	12.73	4	<0.001	**<0.001**	**<0.001**	**<0.001**	**<0.001**	0.063	0.495	0.894	**0.016**	**0.045**	0.572

Statistical results of univariate ANOVA tests for all variables. LSD post-hoc tests reveal significant pairings between dietary group cases.

**Figure 6:**
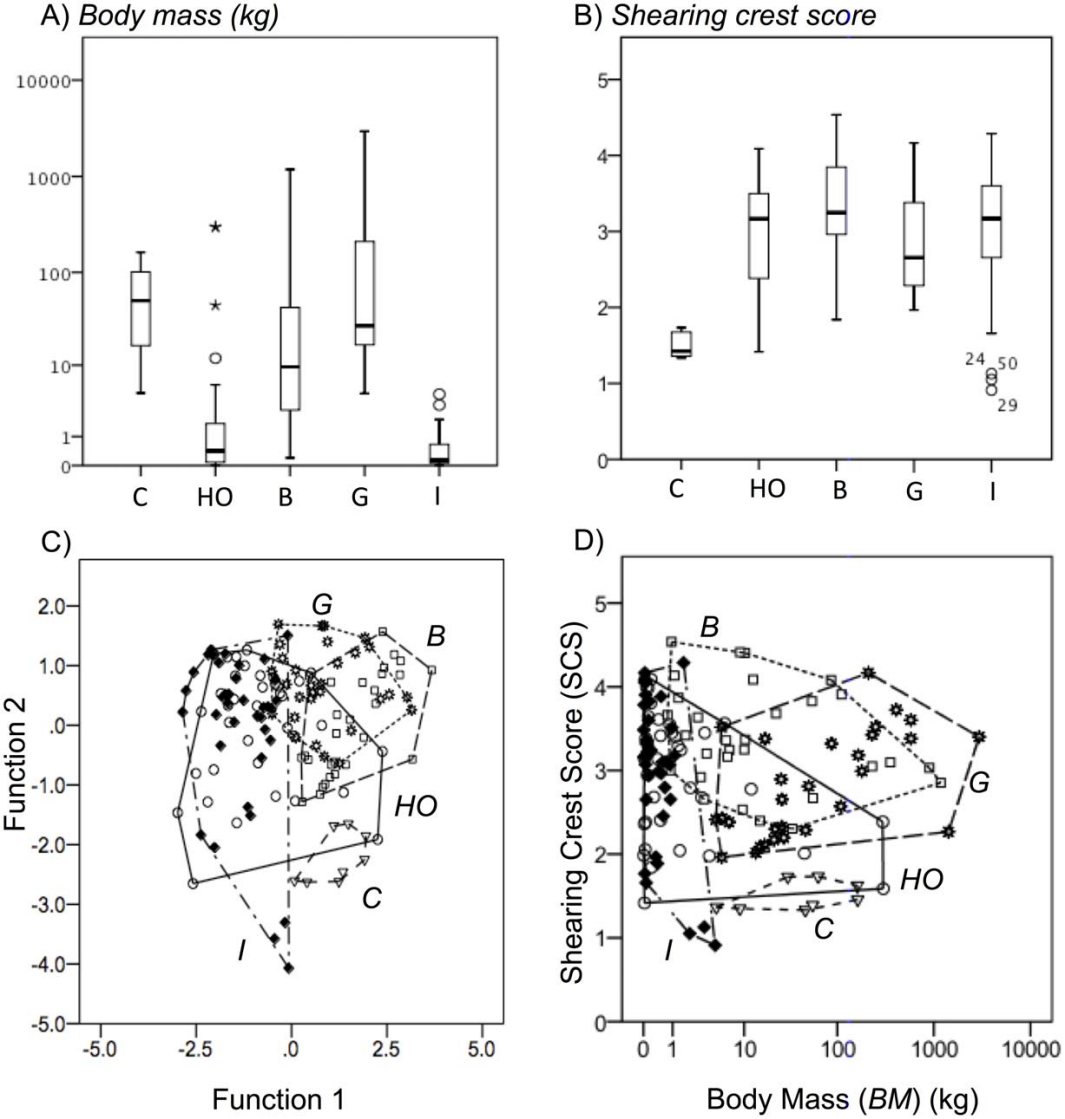
The relative frequencies of body mass and Shearing Crest Score (SCS) within feeding guilds. The distribution of A) body mass (*BM*) and B) Shearing Crest Score (*SCS*) among all specimens and feeding guilds is shown. Box plots show the median (center line), interquartile range (boxes), 1.5 times the interquartile range (whiskers), and outlier points. C) Discriminant function analysis using BM and SCS as dependent variables. HO = Hard-Object Feeders, B = Browsers, G = Grazers, I = Insectivores, C = Carnivores. Function 1 (75.2%) and Function 2 (24.8%) are largely synonymous with *BM/P_s_* and *SCS*, respectively. Wilks’ Lambda = 0.686, Chi-Square = 51.76, p<0.001. The discriminant function coefficients are *BM*_−0.157/0.989_, *SCS*_0.994/0.116_, *P_s_*_0.845/−0.416_, *P_l_*_0.071/−0.166._ D) Bivariate plot, BM vs. SCS, showing polygonal dietary morphospaces. Hulls delineate dietary groups.

Consistent with previously recognized correlations [89], [91], [102], [103], some aspects of diet may be inferred from body size alone. The minimum body size for obligate marsupial and placental browsers is ∼500 g [94], [95]. Obligate insectivores require body sizes smaller than the threshold for browsers due to the size and patchiness of their food sources, with the exception of specialists feeding on colonial insects (i.e. anteaters and aardwolves, not included here due to reduced dentition). As a result, insectivore body mass is significantly different from all four of the other guilds (Table 1), allowing this group to be reliably distinguished (Figure 6A) despite the variability in its microwear patterns.

Calculating a Shearing Crest Score (SCS) for each individual by plotting total shearing crest length against the square root of first molar area (length×width) yielded significant differences between carnivores, hard-object feeders, and browsers/grazers of all orders, marsupials included (Table 1–see also Figure S2 in Supporting Information S1). Carnivores, relying on a maximum force applied to a short cutting blade (carnassial teeth) for prey subdual [45], have significantly shorter SCS than any of the other groups (Figure 6B). Plotting SCS and body mass against one another shows the five feeding guilds occupy polygonal morphospaces of varying overlap (Figure 6D). On the lowest end of the body mass spectrum there is a wide range of SCS scores, reflecting varied diets on the parts of these animals. The insectivores are distinct from high-fiber herbivores, although not from hard-object feeding herbivores: there is considerable overlap in fracture properties in foods used by mammals with small body masses (discussed above), resulting in teeth adapted for similar function in breaking them down. At larger sizes, there is a clear distinction between herbivores (which generally have much more complexly ridged teeth) and carnivores. The three main types of herbivores cannot be confidently distinguished from each other with SCS results, however herbivores, carnivores, and insectivores can be separated out, which is impossible using LDM microwear alone.

**Figure 7:**
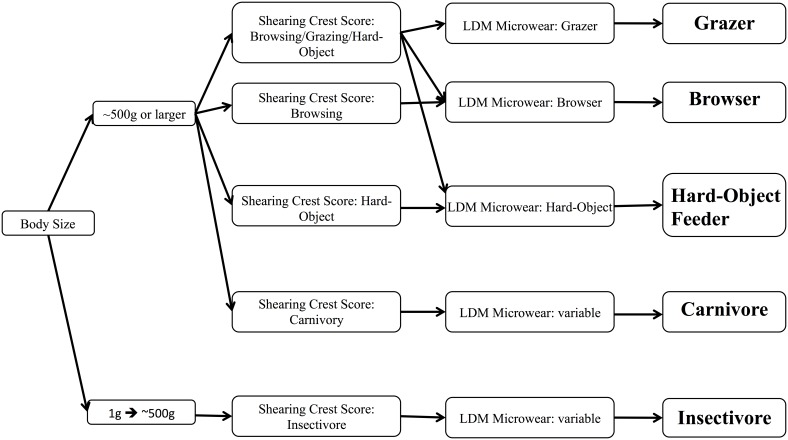
Flow chart representing the path taken toward the assignment of feeding guild using the three different analyses (body size, tooth morphology, LDM microwear) in succession.

Each of the three independently derived lines of evidence (LDM microwear, SCS, and body mass) is informative in different ways for the assignment of diet among the five broad feeding guilds discussed here. None of these techniques on its own is able to discriminate reliably between all five, however the complementary strengths in discriminatory power from all three analytical methods can be taken into account (Figure 7). Body mass is key for certain physiological thresholds related to what food a mammal is able to digest. Once minimum body sizes are surpassed, SCS scores can distinguish carnivores and herbivores. Once herbivory has been indicated by SCS results, LDM microwear analysis can provide assignment to a more specific herbivorous niche.

The following of this algorithm results in correct assignment of dietary guild 92% of the time, determined by re-assigning diet to all specimens used in this study in the context of Figure 7. The uncertainty stems from the overlapping physiological and morphological parameters of insectivores and hard-object feeders, discussed above. These two groups have similar body masses (Figure 6A), and take advantage of patchy resources with similar physical properties (i.e. hard-shelled insects and seeds, both of which are hard and brittle) in an opportunistic fashion; many insectivores supplement their diets with seeds and vice versa. This makes obligate insectivores difficult to distinguish from obligate hard-object feeders of similar size.

## Discussion

Low-magnification microscopy of dental microwear [25], [28], [60], [104], [105], relative shearing crest length [83], [94], [99], [100], [106]–[108], and body mass [90], [94] estimates represent established techniques for the determination of diet in mammals. The results here indicate that, when combined, these characteristics can be used to infer mammalian diets across a remarkable range of phylogeny and animal form. Neither LDM microwear, shearing crest morphology, nor body mass is fully diagnostic as to feeding guild independently: LDM microwear can readily distinguish grazing, hard-object feeding, and browsing, while SCS in combination with body mass delineates herbivores, carnivores, and insectivores. When used in this workflow, however, the techniques outlined here are a powerful tool for the partitioning of dietary habits into the coarse categories evaluated here (Figure 7).

The specimens used in the analysis are drawn from a global sample of extant mammals, collected in different seasons and over several decades. Because microscopic damage records diet over a relatively short period of time [9], the results presented here are noisier than would be derived from sampling a single locality or a single season. That the outcome is nonetheless robust is testimony to the strength of the correlations described. Dietary similarities clearly trump individual morphologies and jaw mechanics, so that distantly related species specializing on a similar set of food objects have comparable shearing crest and microwear parameters.

The method presented here is a powerful tool for assigning a general dietary category to an animal with a truly unknown ecology. Specific dietary distinctions beyond the five categories discussed are outside the scope of this paper, although a variety of studies have used different microwear techniques such as DMTA to subdivide diet (generally within a single mammalian clade) on a much finer scale, which can include looking for evidence of fallback foods and even inter-individual differences [27], [65], [69], [109], [110]. Further work would be needed to know whether the highly specific measurements used as evidence of extremely specific diets in these and other studies have the same universality as the basic scratch and pit counts used here. Such highly specific techniques could certainly be applied after use of the coarse assignment workflow described here to identify appropriate targets for future investigation with finer analyses like DMTA.

Having been tested using extant mammals with known diets and demonstrated to be successful across the mammalian phylogeny, this workflow is also suitable for studies on the evolution and ecology of extinct mammals as well as museum specimens of living species where field study would be difficult owing to the animal’s behavior, habitat, or conservation status. Importantly for the wide application of this diagnostic approach, no significant difference was found between the LDM microwear patterns or SCS distributions of eutherians and metatherians belonging to the same feeding guilds. Marsupial and placental mammals, separated by more than 100 million years of evolution, have different chewing cycles and use their teeth in different ways [76], [111]–[117]. Despite this marsupial and placental mammals belonging to the same dietary niche have statistically indistinguishable microwear and SCS–a remarkable example of the influence of food material properties on oral processing. Thus, evolutionary changes in the diets of the earliest mammals, the ecological selectivity of the end-Cretaceous extinction, and the re-emergence of herbivory in the Paleocene recovery would all be appropriate targets for tooth-based dietary research. Current work is being directed toward the study of the post-Cretaceous mammalian diversification in the western North American interior [118].

## Supporting Information

Supporting Information S1 **Additional data tables and statistical analyses.** This file contains specimen information and data used in the microwear/morphological analyses, as well as the results of statistical analysis of the limited data set.
(DOCX)

## References

[1] Cope ED (1887) The Perissodactyla. Am Nat 21: 985–1007.

[2] Cuozzo FP, Ungar PS, Sauther ML (2012) Primate dental ecology: How teeth respond to the environment. Am J Phys Anthropol 148: 159–162 10.1002/ajpa.22082 22610891

[3] Strait DS, Weber GW, Constantino P, Lucas PW, Richmond BG, et al (2012) Microwear, mechanics and the feeding adaptations of Australopithecus africanus. J Hum Evol 62: 165–168 10.1016/j.jhevol.2011.10.006 22130183

[4] Schulz E, Piotrowski V, Clauss M, Mau M, Merceron G, et al (2013) Dietary abrasiveness is associated with variability of microwear and dental surface texture in rabbits. PLoS ONE 8: e56167 10.1371/journal.pone.0056167.s001 23405263 PMC3566079

[5] Grine FE, Sponheimer M, Ungar PS, Lee-Thorp J, Teaford MF (2012) Dental microwear and stable isotopes inform the paleoecology of extinct hominins. Am J Phys Anthropol 148: 285–317 10.1002/ajpa.22086 22610903

[6] Mihlbachler MC, Solounias N (2006) Coevolution of tooth crown height and diet in oreodonts (Merycoidodontidae, Artiodactyla) examined with phylogenetically independent contrasts. J Mammal Evol 13: 11–36 10.1007/s10914-005-9001-3

[7] Mainland I (2003) Dental microwear in grazing and browsing Gotland sheep (*Ovis aries*) and its implications for dietary reconstruction. J Archaeol Sci 30: 1513–1527.

[8] Mainland I (2006) Pastures lost? A dental microwear study of ovicaprine diet and management in Norse Greenland. J Archaeol Sci 33: 238–252 10.1016/j.jas.2005.07.013

[9] Teaford M, Oyen O (1989) In vivo and in vitro turnover in dental microwear. Am J Phys Anthropol 80: 447–460. 2513725 10.1002/ajpa.1330800405

[10] Teaford M, Maas M, Simons E (1996) Dental microwear and microstructure in early Oligocene primates from the Fayum, Egypt: Implications for diet. Am J Phys Anthropol 101: 527–543. 9016366 10.1002/(SICI)1096-8644(199612)101:4<527::AID-AJPA7>3.0.CO;2-S

[11] Teaford M (1992) Dental microwear and diet in Venezuelan primates. Am J Phys Anthropol 88: 347–364. 1642321 10.1002/ajpa.1330880308

[12] Calandra I, Schulz E, Pinnow M, Krohn S, Kaiser TM (2012) Teasing apart the contributions of hard dietary items on 3D dental microtextures in primates. J Hum Evol 63: 85–98 10.1016/j.jhevol.2012.05.001 22705031

[13] Ramdarshan A, Merceron G, Tafforeau P, Marivaux L (2010) Dietary reconstruction of the Amphipithecidae (Primates, Anthropoidea) from the Paleogene of South Asia and paleoecological implications. J Hum Evol 59: 96–108 10.1016/j.jhevol.2010.04.007 20510435

[14] Galbany J, Estebaranz F, Martínez LM, Pérez-Pérez A (2009) Buccal dental microwear variability in extant African Hominoidea: taxonomy versus ecology. Primates 50: 221–230 10.1007/s10329-009-0139-0 19296198

[15] Godfrey L, Semprebon G, Jungers W, Sutherland M, Simons E, et al (2004) Dental use wear in extinct lemurs: evidence of diet and niche differentiation. J Hum Evol 47: 145–169 10.1016/j.jhevol.2004.06.003 15337413

[16] Godfrey LR, Semprebon GM, Schwartz GT, Burney DA, Jungers WL, et al (2005) New insights into old lemurs: the trophic adaptations of the archaeolemuridae. Int J Primatol 26: 825–854 10.1007/s10764-005-5325-3

[17] Green J (2009) Dental microwear in the orthodentine of the Xenarthra (Mammalia) and its use in reconstructing the palaeodiet of extinct taxa: the case study of *Nothrotheriops shastensis* (Xenarthra, Tardigrada, Nothrotheriidae. Zool J Lin Soc-Lond 156: 201–222.

[18] Rivals F, Semprebon G, Lister A (2012) An examination of dietary diversity patterns in Pleistocene proboscideans (Mammuthus, Palaeoloxodon, and Mammut) from Europe and North America as revealed by dental microwear. Quatern Int 255: 188–195 10.1016/j.quaint.2011.05.036

[19] Green J, Semprebon G, Solounias N (2005) Reconstructing the palaeodiet of Florida *Mammut americanum* via low-magnification stereomicroscopy. Palaeogeogr Palaeocl 223: 34–48.

[20] DeSantis LRG, Schubert BW, Scott JR, Ungar PS (2012) Implications of diet for the extinction of saber-toothed cats and American lions. PLoS ONE 7(12): e52453 10.1371/journal.pone.0052453.s002 23300674 PMC3530457

[21] Ungar PS, Scott JR, Schubert BW, Stynder DD (2010) Carnivoran dental microwear textures: comparability of carnassial facets and functional differentiation of postcanine teeth. Mammalia 74: 219–224 10.1515/MAMM.2010.015

[22] Schubert BW, Ungar PS, Desantis LRG (2010) Carnassial microwear and dietary behaviour in large carnivorans. J Zool 280: 257–263 10.1111/j.1469-7998.2009.00656.x

[23] Strait S (1993) Molar microwear in extant small-bodied faunivorous mammals - an analysis of feature density and pit frequency. Am J Phys Anthropol 92: 63–79. 8238292 10.1002/ajpa.1330920106

[24] Rivals F, Athanassiou A (2008) Dietary adaptations in an ungulate community from the late Pliocene of Greece. Palaeogeogr Palaeocl 265: 134–139 10.1016/j.palaeo.2008.05.001

[25] Rivals F, Semprebon GM (2011) Dietary plasticity in ungulates: Insight from tooth microwear analysis. Quatern Int 245: 279–284 10.1016/j.quaint.2010.08.001

[26] Merceron G, Blondel C, Viriot L, Koufos GD, de Bonis L (2007) Dental microwear analysis of bovids from the Vallesian (late Miocene) of Axios Valley in Greece: reconstruction of the habitat of *Ouranopithecus macedoniensis* (Primates, Hominoidea). Geodiversitas 29: 421–433.

[27] Merceron G, Viriot L, Blondel C (2004) Tooth microwear pattern in roe deer (*Capreolus capreolus L.*) from Chize (Western France) and relation to food composition. Small Ruminant Res 53: 125–132 10.1016/j.smallrumers.2003.10.002

[28] Solounias N, Semprebon G (2002) Advances in the reconstruction of ungulate ecomorphology with application to early fossil equids. Am Mus Novit 3366: 1–49.

[29] Schulz E, Calandra I, Kaiser TM (2013) Feeding ecology and chewing mechanics in hoofed mammals: 3D tribology of enamel wear. Wear 300(1): 169–179.

[30] Gomes Rodrigues H, Merceron G, Viriot L (2009) Dental microwear patterns of extant and extinct Muridae (Rodentia, Mammalia): ecological implications. Naturwissenschaften 96: 537–542 10.1007/s00114-008-0501-x 19127354

[31] Townsend KEB, Croft DA (2008) Enamel microwear in caviomorph rodents. J Mammal 89: 730–743.

[32] Charles C, Jaeger J-J, Michaux J, Viriot L (2006) Dental microwear in relation to changes in the direction of mastication during the evolution of Myodonta (Rodentia, Mammalia). Naturwissenschaften 94: 71–75 10.1007/s00114-006-0161-7 17016685

[33] Nelson S, Badgley C, Zakem E (2005) Microwear in modern squirrels in relation to diet. Palaeontologia Electronica 8: 1–15.

[34] Purnell M, Seehausen O, Galis F (2012) Quantitative three-dimensional microtextural analyses of tooth wear as a tool for dietary discrimination in fishes. J R Soc Interface 9: 2225–2233 10.1002/sca.20181 22491979 PMC3405762

[35] Purnell MA, Crumpton N, Gill PG, Jones G, Rayfield EJ (2013) Within-guild dietary discrimination from 3-D textural analysis of tooth microwear in insectivorous mammals. J Zool 291: 249–257. 10.1111/jzo.12068 PMC4296236 25620853

[36] Prideaux GJ, Ayliffe LK, DeSantis LRG, Schubert BW, Murray PF, et al (2009) Extinction implications of a chenopod browse diet for a giant Pleistocene kangaroo. P Natl Acad Sci USA 106: 11646–11650. 10.1073/pnas.0900956106 PMC2710660 19556539

[37] Mainland I (2000) A dental microwear study of seaweed-eating and grazing sheep from Orkney. Int J Osteoarchaeol 10: 93–107.

[38] Teaford M (1991) Dental microwear in live, wild-trapped *Alouatta palliata* from Costa Rica. Am J Phys Anthropol 85: 313–319. 1897604 10.1002/ajpa.1330850310

[39] Lucas PW, Omar R, Al-Fadhalah K, Almusallam AS, Henry AG, et al (2012) Mechanisms and causes of wear in tooth enamel: implications for hominin diets. J R Soc Interface 10: 20120923–20120923 10.1007/s10856-010-3988-6 PMC3565742 23303220

[40] Ungar P, Ungar P, Teaford M, Teaford M, Glander K, et al (1995) Dust accumulation in the canopy: a potential cause of dental microwear in primates. Am J Phys Anthropol 97: 93–99. 7653510 10.1002/ajpa.1330970202

[41] Danielson D, Reinhard K (1998) Human dental microwear caused by calcium oxalate phytoliths in prehistoric diet of the lower Pecos region, Texas. Am J Phys Anthropol 107: 297–304. 9821494 10.1002/(SICI)1096-8644(199811)107:3<297::AID-AJPA6>3.0.CO;2-M

[42] Sanson GD, Kerr SA, Gross KA (2007) Do silica phytoliths really wear mammalian teeth? J Archaeol Sci 34: 526–531 10.1016/j.jas.2006.06.009

[43] van Valkenburgh B, Teaford M (1990) Molar microwear and diet in large carnivores: inferences concerning diet in the sabretooth cat, Smilodon fatalis. J Zool 222: 319–340.

[44] Strait S (1997) Tooth use and the physical properties of food. Evol Anthropol 5: 199–211.

[45] Lucas PW (2004) Dental functional morphology. Cambridge: Cambridge Univ Press. 355.

[46] Clissold FJ (2011) The biomechanics of chewing and plant fracture: Mechanisms and implications. Adv Insect Physiol 34: 317–372 10.1016/S0065-2806(07)34006-X

[47] Vincent J (1990) Fracture properties of plants. Adv Bot Res 17: 235–287.

[48] Wright W, Vincent J (1996) Herbivory and the mechanics of fracture in plants. Biol Rev 71: 401–413.

[49] Lucas P (2000) Mechanical defenses to herbivory. Annal Bot-London 86: 913–920 10.1006/anbo.2000.1261

[50] Lucas PW, Prinz JF, Agrawal KR, Bruce IC (2002) Food physics and oral physiology. Food Qual Prefer 13: 203–213.

[51] Evans AR, Sanson GD (2003) The tooth of perfection: functional and spatial constraints on mammalian tooth shape. Biol J Linn Soc 78: 173–191.

[52] Teaford M (1988) A review of dental microwear and diet in modern mammals. Scanning Microscopy 2: 1149–1166. 3041572

[53] Strait S (1993) Molar microwear in extant small-bodied faunivorous mammals: An analysis of feature density and pit frequency. Am J Phys Anthropol 92: 63–79. 8238292 10.1002/ajpa.1330920106

[54] Ungar P (1995) A semiautomated image analysis procedure for the quantification of dental microwear II. Scanning 17: 57–59. 7704317 10.1002/sca.4950170108

[55] Walker A, Hoeck H, Perez L (1978) Microwear of mammalian teeth as an indicator of diet. Science 201: 908–910. 684415 10.1126/science.684415

[56] Grine F (1981) Trophic differences between “gracile” and “robust” Australopithecines: a scanning electron microscope analysis of occlusal events. S Afr J Sci 77: 203–230.

[57] Teaford M (1984) Quantitative differences in dental microwear between primate species with different diets and a comment on the presumed diet of Sivapithecus. Am J Phys Anthropol 64: 191–200. 6380302 10.1002/ajpa.1330640213

[58] Grine F, Ungar P, Teaford M (2002) Error rates in dental microwear quantification using scanning electron microscopy. Scanning 24: 144–153. 12074496 10.1002/sca.4950240307

[59] Galbany J, Martinez L, Amor HL (2005) Error rates in buccal-dental microwear quantification using scanning electron microscopy. Scanning 27: 23–29. 15712754 10.1002/sca.4950270105

[60] Semprebon G, Godfrey L, Solounias N, Sutherland M, Jungers W (2004) Can low-magnification stereomicroscopy reveal diet? J Hum Evol 47: 115–144 10.1016/j.jhevol.2004.06.004 15337412

[61] Merceron G, Blondel C, Brunet M, Sen S, Solounias N, et al (2004) The Late Miocene paleoenvironment of Afghanistan as inferred from dental microwear in artiodactyls. Palaeogeogr Palaeocl 207: 143–163 10.1016/j.palaeo.2004.02.008

[62] Merceron G (2005) A new method of dental microwear analysis: Application to extant primates and *Ouranopithecus macedoniensis* (Late Miocene of Greece). Palaios 20: 551–561 10.2110/palo.2004.p04-17

[63] Gordon K (1988) A review of methodology and quantification in dental microwear analysis. Scanning Microscopy 2: 1139–1147. 3041571

[64] Mihlbachler M, Beatty B, Caldera-Siu A, Chan D, Lee R (2012) Error rates and observer bias in dental microwear analysis using light microscopy. Palaeontologia Electronica 15: 1–22.

[65] Scott RS, Ungar PS, Bergstrom TS, Brown CA, Grine FE, et al (2005) Dental microwear texture analysis shows within-species diet variability in fossil hominins. Nature 436: 693–695 10.1038/nature03822 16079844

[66] Scott RS, Ungar PS, Bergstrom TS, Brown CA, Childs BE, et al (2006) Dental microwear texture analysis: technical considerations. J Hum Evol 51: 339–349. 16908052 10.1016/j.jhevol.2006.04.006

[67] Ungar P, Grine F, Teaford M, Zaatari El S (2006) Dental microwear and diets of African early Homo. J Hum Evol 50: 78–95 10.1016/j.jhevol.2005.08.007 16226788

[68] DeSantis L, Scott JR, Schubert BW, Donohue SL (2013) Direct comparisons of 2D and 3D dental microwear proxies in extant herbivorous and carnivorous mammals. PLoS ONE. doi:10.1371/journal.pone.0071428.s006. 10.1371/journal.pone.0071428 PMC3735535 23936506

[69] Merceron G, Escarguel G, Angibault J-M, Verheyden-Tixier H (2010) Can dental microwear textures record inter-individual dietary variations? PLoS ONE 5: e9542 10.1371/journal.pone.0009542.s002 20209051 PMC2832010

[70] Krueger KL, Scott JR, Kay RF, Ungar PS (2008) Technical note: Dental microwear textures of “Phase I” and “Phase II” facets. Am J Phys Anthropol 137: 485–490 10.1002/ajpa.20928 18785631

[71] Dewar EW (2004) Microwear of carnivorous mammals described by low-magnification dental stereomicroscopy. J Vertebr Paleontol 24: 52A.

[72] Ungar PS (2010) Mammal teeth. Baltimore: Johns Hopkins University Press. 304.

[73] Sanson G (1989) Morphological adaptations of teeth to diets and feeding in the Macropodoidea. In: Grigg G, Jarman P and Hume I, editors. Kangaroos, wallabies, and rat-kangaroos. New South Wales: Surrey, Beatty & Sons Pty Limited. 151–168.

[74] Janis C, Fortelius M (1988) On the means whereby mammals achieve increased functional durability of their dentitions, with special reference to limiting factors. Biol Rev 63: 197–230. 3042033 10.1111/j.1469-185x.1988.tb00630.x

[75] Fortelius M (1990) The mammalian dentition, a tangled view. Neth J Zool 40: 312–328.

[76] Herring S (1993) Functional morphology of mammalian mastication. Am Zool 33: 289–299.

[77] Friscia AR, Van Valkenburgh B, Biknevicius A (2007) An ecomorphological analysis of extant small carnivorans. J Zool 272: 82–100.

[78] Santana S, Strait S (2011) The better to eat you with: functional correlates of tooth structure in bats. Funct Ecology 25: 839–847.

[79] Jernvall J, Hunter J, Fortelius M (1996) Molar tooth diversity, disparity, and ecology in cenozoic ungulate radiations. Science 274: 1489–1492. 8929401 10.1126/science.274.5292.1489

[80] Van Valkenburgh B (1989) Carnivore dental adaptations and diet, a study of trophic diversity within guilds. In: Gittleman, JL editor. Carnivore behavior, ecology and evolution Vol. 1. Ithaca: Cornell University Press. 410–436.

[81] Sacco T, Van Valkenburgh B (2004) Ecomorphological indicators of feeding behaviour in bears (Carnivora: Ursidae). J Zool 263: 41–54.

[82] Wilson GP, Evans AR, Corfe IJ, Smits PD, Fortelius M, et al (2012) Adaptive radiation of multituberculate mammals before the extinction of dinosaurs. Nature 483: 457–460 10.1038/nature10880 22419156

[83] Strait S (1993) Differences in occlusal morphology and molar size in frugivores and faunivores. J Hum Evol 25: 471–484.

[84] Evans AR, Wilson GP, Fortelius M, Jernvall J (2007) High-level similarity of dentitions in carnivorans and rodents. Nature 445: 78–81 10.1038/nature05433 17167416

[85] Fritz J, Hummel J, Kienzle E, Arnold C, Nunn C, et al (2009) Comparative chewing efficiency in mammalian herbivores. Oikos 118: 1623–1632 10.1111/j.1600-0706.2009.17807.x

[86] Clauss M, Hummel J (2005) The digestive performance of mammalian herbivores: why big may not be that much better. Mammal Rev 35: 174–187.

[87] Ungar PS, Grine FE, Teaford MF (2008) Dental microwear and diet of the Plio-Pleistocene hominin *Paranthropus boisei* . PLoS ONE 3: e2044 10.1371/journal.pone.0002044.s005 18446200 PMC2315797

[88] Legendre S (1986) Analysis of mammalian communities from the late Eocene and Oligocene of southern France. Paleovertebrata 16: 191–212.

[89] MacFadden B, Damuth J (1990) Introduction: Body size and its estimation. In: MacFadden B, Damuth J, editors. Body size in mammalian paleobiology: Estimation and biological implications. Cambridge: Cambridge University Press. 1–10.

[90] Clauss M, Streich WJ, Nunn CL, Ortmann S, Hohmann G, et al (2008) The influence of natural diet composition, food intake level, and body size on ingesta passage in primates. Comp Biochem Phys A 150: 274–281 10.1016/j.cbpa.2008.03.012 18450489

[91] Clauss M, Kaiser T, Hummel J (2008) The morphophysiological adaptations of browsing and grazing mammals. In: Gordon I, Prins H, editors. The ecology of browsing and grazing. Springer Berlin Heidelberg. 47–88.

[92] Clauss M, Jürgen Streich W, Schwarm A, Ortmann S, Hummel J (2007) The relationship of food intake and ingesta passage predicts feeding ecology in two different megaherbivore groups. Oikos 116: 209–216 10.1111/j.2006.0030-1299.15461.x

[93] McNab BK (2002) The physiological ecology of vertebrates: A view from energetics. Ithaca: Cornell University Press. 576.

[94] Kay R (1975) Functional adaptations of primate molar teeth. Am J Phys Anthropol 43: 195–215. 810034 10.1002/ajpa.1330430207

[95] Hogue AS, ZiaShakeri S (2010) Molar crests and body mass as dietary indicators in marsupials. Aust J Zool 58: 56–68.

[96] Bininda-Emonds ORP, Cardillo M, Jones KE, Macphee RDE, Beck RMD, et al (2007) The delayed rise of present-day mammals. Nature 446: 507–512 10.1038/nature05634 17392779

[97] Nowak RM (1999) Walker’s Mammals of the World. 9 ed. Baltimore: Johns Hopkins University Press. 1936.

[98] Goillot C, Blondel C, Peigné S (2009) Relationships between dental microwear and diet in Carnivora (Mammalia) – Implications for the reconstruction of the diet of extinct taxa. Palaeogeogr Palaeocl 271: 13–23 10.1016/j.palaeo.2008.09.004

[99] Ungar PS, Kay RF (1995) The dietary adaptations of European Miocene catarrhines. P Natl Acad Sci Usa 92: 5479–5481. 10.1073/pnas.92.12.5479 PMC41718 7777533

[100] Strait S (1993) Molar morphology and food texture among small-bodied insectivorous mammals. J Mammal 74: 391–402.

[101] Smith F, Lyons S, Ernest S, Jones K, Kaufman D, et al (2003) Body mass of late quaternary mammals. Ecology 84: 3403–3403.

[102] Demment MW, Van Soest PJ (2009) A nutritional explanation for body-size patterns of ruminant and nonruminant herbivores. Am Nat: 641–672.

[103] Clauss M, Frey R, Kiefer B, Lechner-Doll M, Loehlein W, et al (2003) The maximum attainable body size of herbivorous mammals: morphophysiological constraints on foregut, and adaptations of hindgut fermenters. Oecologia 136: 14–27 10.1007/s00442-003-1254-z 12712314

[104] Baker G, Jones L, Wardrop ID (1959) Cause of wear in sheeps’ teeth. Nature 184: 1583–1584. 13795990 10.1038/1841583b0

[105] Rivals F, Solounias N (2007) Differences in tooth microwear of populations of caribou (*Rangifer tarandus*, Ruminantia, Mammalia) and implications to ecology, migration, glaciations and dental evolution. J Mammal Evol 14: 182–192 10.1007/s10914-007-9044-8

[106] Strait S (2001) Dietary reconstruction of small-bodied omomyoid primates. J Vertebr Paleontol 21: 322–334.

[107] Anthony M, Kay R (1993) Tooth form and diet in ateline and alouattine primates: reflections on the comparative method. Am J Sci 293A: 356–382.

[108] Kay R, Sussman RW, Tattersall I (1978) Dietary and dental variations in genus Lemur, with comments concerning dietary-dental correlations among Malagasy primates. Am J Phys Anthropol 49: 119–127. 98050 10.1002/ajpa.1330490118

[109] Semprebon GM, Sise PJ, Coombs MC (2010) Potential bark and fruit browsing as revealed by stereomicrowear analysis of the peculiar clawed herbivores known as chalicotheres (Perissodactyla, Chalicotherioidea). J Mammal Evol 18: 33–55 10.1007/s10914-010-9149-3

[110] Rafferty K, Teaford M, Jungers W (2002) Molar microwear of subfossil lemurs: improving the resolution of dietary inferences. J Hum Evol 43: 645–657 10.1053/jhev.2002.0592 12457853

[111] Crompton AW, Barnet J, Lieberman DE, Owerkowicz T, Skinner J, et al (2008) Control of jaw movements in two species of macropodines (*Macropus eugenii* and *Macropus rufus*). Comp Biochem Phys A 150: 109–123 10.1016/j.cbpa.2007.10.015 18065250

[112] Crompton AW, Owerkowicz T, Skinner J (2010) Masticatory motor pattern in the koala (*Phascolarctos cinereus*): a comparison of jaw movements in marsupial and placental herbivores. J Exp Zool 313A: 564–578 10.1002/jez.628 20683866

[113] Crompton AW (2011) Masticatory motor programs in Australian herbivorous mammals: Diprotodontia. Integr Comp Biol 51: 271–281 10.1093/icb/icr028 21700567

[114] Crompton A, Lieberman D, Owerkowicz T, Baudinette R, Skinner J (2008) Motor control of masticatory movements in the Southern hairy-nosed wombat. In: Vinyard C, editor. Primate Craniofacial Function and Biology. New York: Springer. 83–111.

[115] Rensberger J, Forsten A, Fortelius M (1984) Functional Evolution of the Cheek Tooth Pattern and Chewing Direction in Tertiary Horses. Paleobiology 10: 439–452.

[116] Ross CF, Dharia R, Herring SW, Hylander WL, Liu Z-J, et al (2007) Modulation of mandibular loading and bite force in mammals during mastication. J Exp Biol 210: 1046–1063. 17337717 10.1242/jeb.02733

[117] Hiiemae K, Kay R (1972) Trends in evolution of primate mastication. Nature 240: 486–487. 4565949 10.1038/240486a0

[118] Christensen H (2012) PhD Thesis: Mammalian adaptation to herbivory in the aftermath of the KT extinction. Chicago, IL: The University of Chicago Press.

