# Supplementary material for: Similar Associations of Tooth Microwear and Morphology Indicate Similar Diet across Marsupial and Placental Mammals
Source: PLoS One. 2014 Aug 6;9(8):e102789. doi: 10.1371/journal.pone.0102789 (PMC4123885; doi:10.1371/journal.pone.0102789)
Supplement: Supporting Information S1 — Additional data tables and statistical analyses. This file contains specimen information and data used in the microwear/morphological analyses, as well as the results of statistical analysis of the limited data set. (DOCX) [file pone.0102789.s001.docx]

**SUPPORTING INFORMATION**

**SECTION 1**

*Table S1***.** List of all individuals included in the microwear analysis. Feature counts listed are averages; up to four measurements were taken per individual (single facet, protocone), size permitting; variation between measurements is reflected in the standard deviation values. A standard deviation value of zero indicates that only a single measurement was possible due to small tooth size. FMNH = Field Museum of Natural History, Chicago IL; AMNH= American Museum of Natural History, New York NY. Under Phylogeny, E= Eutherian, M=Metatherian; Diet Category, I=Insectivore, C=Carnivore, G=Grazer, B=Browser, H=Hard-Object Feeder.

| Phylogeny | Diet Category | Order | Specimen Number | Species | Continent | Fine Scratches | Fine STDEV | Coarse Scratches | Coarse STDEV | Total Scratches | Total Scratch STDEV | Small Pits | Small STDEV | Large Pits | Large STDEV | Total Pits | Total Pit STDEV |
| --- | --- | --- | --- | --- | --- | --- | --- | --- | --- | --- | --- | --- | --- | --- | --- | --- | --- |
| E | I | Afrosoricidae | FMNH 159728 | Echinops telfairi | Africa | 28.00 | 2.83 | 3.50 | 2.12 | 31.50 | 4.95 | 35.50 | 6.36 | 11.00 | 1.41 | 46.50 | 4.95 |
| E | I | Afrosoricidae | FMNH 159729 | Echinops telfairi | Africa | 31.00 | 0.00 | 2.00 | 0.00 | 33.00 | 0.00 | 36.00 | 0.00 | 12.00 | 0.00 | 48.00 | 0.00 |
| E | I | Afrosoricidae | FMNH 158730 | Echinops telfairi | Africa | 30.00 | 0.00 | 4.00 | 0.00 | 34.00 | 0.00 | 32.00 | 0.00 | 12.00 | 0.00 | 44.00 | 0.00 |
| E | I | Afrosoricidae | FMNH 176212 | Geogale aurita | Africa | 18.00 | 0.00 | 5.00 | 0.00 | 23.00 | 0.00 | 24.00 | 0.00 | 19.00 | 0.00 | 43.00 | 0.00 |
| E | I | Afrosoricidae | FMNH 154474 | Tenrec ecaudatus | Africa | 42.00 | 0.00 | 0.00 | 0.00 | 42.00 | 0.00 | 54.00 | 0.00 | 18.00 | 0.00 | 72.00 | 0.00 |
| E | I | Afrosoricidae | FMNH 156336 | Tenrec ecaudatus | Africa | 37.00 | 0.00 | 1.00 | 0.00 | 38.00 | 0.00 | 44.00 | 0.00 | 13.00 | 0.00 | 57.00 | 0.00 |
| E | I | Afrosoricidae | FMNH 156337 | Tenrec ecaudatus | Africa | 34.00 | 0.00 | 3.50 | 2.12 | 37.50 | 0.71 | 45.00 | 11.31 | 19.50 | 0.71 | 64.50 | 10.61 |
| E | B | Artiodactyla | FMNH 15571 | Alces alces americanus | North America | 20.00 | 0.00 | 0.00 | 0.00 | 20.00 | 0.00 | 24.00 | 0.00 | 13.00 | 0.00 | 37.00 | 0.00 |
| E | B | Artiodactyla | FMNH 35258 | Alces alces gigas | North America | 15.50 | 4.80 | 1.25 | 1.50 | 16.75 | 3.50 | 23.25 | 4.92 | 10.25 | 2.87 | 33.50 | 6.14 |
| E | G | Artiodactyla | FMNH 21151 | Bison bison bison | North America | 16.67 | 4.73 | 12.33 | 6.30 | 29.00 | 3.00 | 17.33 | 2.08 | 12.67 | 2.08 | 30.00 | 3.61 |
| E | G | Artiodactyla | FMNH 21512 | Bison bison bison | North America | 20.75 | 6.02 | 13.25 | 5.58 | 34.00 | 8.83 | 14.00 | 2.16 | 10.25 | 1.89 | 24.25 | 2.75 |
| E | B | Artiodactyla | FMNH 92923 | Capra hircus aegagrus | Asia | 10.00 | 1.41 | 2.00 | 1.97 | 12.00 | 2.83 | 17.00 | 1.15 | 16.50 | 4.80 | 33.50 | 5.92 |
| E | H | Artiodactyla | FMNH 26916 | Cephalophus natalensis robertsi | Africa | 20.75 | 3.30 | 6.00 | 2.22 | 26.75 | 2.63 | 43.75 | 2.22 | 22.25 | 9.54 | 66.00 | 8.37 |
| E | B | Artiodactyla | FMNH 34423 | Giraffa camelopardalis angolensis | Africa | 12.00 | 0.82 | 1.25 | 1.32 | 13.25 | 1.71 | 17.75 | 2.50 | 11.75 | 4.50 | 29.50 | 2.52 |
| E | B | Artiodactyla | FMNH 34425 | Giraffa camelopardalis angolensis | Africa | 10.25 | 2.63 | 3.25 | 2.71 | 13.50 | 3.11 | 17.50 | 5.07 | 16.00 | 2.45 | 33.50 | 7.23 |
| E | G | Artiodactyla | FMNH 127871 | Hippopotamus amphibius | Africa | 33.00 | 3.16 | 5.75 | 2.26 | 38.75 | 2.87 | 24.75 | 4.27 | 10.25 | 1.71 | 35.00 | 4.76 |
| E | G | Artiodactyla | FMNH 1091 | Kobus ellipsiprymnus ellipsiprymnus | Africa | 28.00 | 2.94 | 9.50 | 1.76 | 37.50 | 3.11 | 21.00 | 5.23 | 15.50 | 0.58 | 36.50 | 5.26 |
| E | G | Artiodactyla | FMNH 20662 | Kobus ellipsiprymnus harnieri | Africa | 15.75 | 0.96 | 10.25 | 3.12 | 26.00 | 0.82 | 26.00 | 3.65 | 17.25 | 2.63 | 43.25 | 5.68 |
| E | G | Artiodactyla | FMNH 18999 | Odocoileus hemionus fuliginatus | North America | 19.00 | 1.00 | 14.33 | 3.52 | 33.33 | 2.52 | 18.33 | 6.51 | 7.33 | 1.53 | 25.67 | 7.64 |
| E | B | Artiodactyla | FMNH 26066 | Okapia johnstoni | Africa | 8.50 | 2.38 | 0.00 | 0.00 | 8.50 | 2.38 | 15.00 | 3.46 | 9.25 | 5.12 | 24.25 | 6.45 |
| E | G | Artiodactyla | FMNH 57255 | Ovis aries aries | Asia | 23.00 | 2.45 | 7.50 | 4.31 | 30.50 | 3.42 | 22.50 | 2.38 | 13.00 | 3.16 | 35.50 | 4.20 |
| E | G | Artiodactyla | FMNH 92928 | Ovis aries aries | Asia | 22.50 | 3.11 | 9.00 | 4.28 | 31.50 | 0.58 | 14.50 | 1.29 | 12.25 | 1.89 | 26.75 | 2.36 |
| E | G | Artiodactyla | FMNH 140855 | Syncerus caffer caffer | Africa | 23.50 | 5.45 | 12.25 | 6.14 | 35.75 | 8.54 | 14.50 | 4.36 | 13.50 | 3.87 | 28.00 | 8.04 |
| E | H | Artiodactyla | FMNH 38013 | Tragulus javanicus affinis | Asia | 19.50 | 6.36 | 4.50 | 2.12 | 24.00 | 7.07 | 44.00 | 7.07 | 22.50 | 2.12 | 66.50 | 4.95 |
| E | H | Artiodactyla | FMNH 33523 | Tragulus javanicus klossi | Asia | 18.00 | 1.41 | 13.00 | 2.83 | 31.00 | 4.24 | 33.50 | 0.71 | 24.50 | 4.95 | 58.00 | 4.24 |
| E | G | Carnivora | FMNH 34258 | Ailuropoda melanoleuca | Asia | 27.50 | 6.81 | 5.00 | 2.83 | 32.50 | 5.00 | 13.75 | 5.19 | 14.50 | 4.04 | 28.25 | 5.85 |
| E | G | Carnivora | FMNH 39514 | Ailuropoda melanoleuca | Asia | 25.25 | 5.32 | 4.50 | 2.21 | 29.75 | 3.50 | 18.50 | 4.51 | 10.25 | 4.43 | 28.75 | 8.06 |
| E | I | Carnivora | FMNH 32556 | Chrotogale owstoni | Asia | 22.00 | 8.49 | 4.50 | 2.12 | 26.50 | 10.61 | 32.00 | 5.66 | 18.50 | 2.12 | 50.50 | 3.54 |
| E | C | Carnivora | FMNH 27007 | Crocuta crocuta | Africa | 25.75 | 4.57 | 6.00 | 2.94 | 31.75 | 5.26 | 35.50 | 5.45 | 9.00 | 4.83 | 44.50 | 9.26 |
| E | C | Carnivora | FMNH 104022 | Crocuta crocuta | Africa | 15.50 | 3.54 | 7.50 | 0.71 | 23.00 | 4.24 | 20.50 | 0.00 | 4.50 | 0.00 | 25.00 | 2.83 |
| E | C | Carnivora | FMNH 135072 | Crocuta crocuta | Africa | 17.67 | 4.51 | 4.00 | 2.00 | 21.67 | 2.49 | 26.33 | 1.53 | 9.33 | 1.15 | 35.67 | 2.52 |
| E | C | Carnivora | FMNH 27026 | Felis silvestris ocreata | Africa | 27.00 | 0.00 | 5.00 | 0.00 | 32.00 | 0.00 | 27.00 | 0.00 | 9.00 | 0.00 | 36.00 | 0.00 |
| E | C | Carnivora | FMNH 32941 | Felis silvestris ocreata | Africa | 18.00 | 1.41 | 1.00 | 1.41 | 19.00 | 2.83 | 49.50 | 12.02 | 29.00 | 12.73 | 78.50 | 0.71 |
| E | C | Carnivora | FMNH 93872 | Felis silvestris ugandae | Africa | 17.00 | 0.00 | 3.00 | 0.00 | 20.00 | 0.00 | 29.00 | 0.00 | 29.00 | 0.00 | 58.00 | 0.00 |
| E | C | Carnivora | FMNH 93876 | Felis silvestris ugandae | Africa | 21.00 | 0.00 | 0.00 | 0.00 | 21.00 | 0.00 | 23.00 | 0.00 | 11.00 | 0.00 | 34.00 | 0.00 |
| E | C | Carnivora | FMNH 93877 | Felis silvestris ugandae | Africa | 27.00 | 1.41 | 5.00 | 1.41 | 32.00 | 2.83 | 26.00 | 1.41 | 22.50 | 2.12 | 48.50 | 3.54 |
| E | I | Carnivora | FMNH 33465 | Hemigalus derbyanus boiei | Asia | 17.00 | 2.83 | 3.50 | 1.41 | 20.50 | 4.95 | 37.00 | 4.24 | 18.50 | 3.54 | 55.50 | 7.78 |
| E | I | Carnivora | FMNH 68723 | Hemigalus derbyanus boiei | Asia | 26.50 | 0.71 | 4.50 | 0.71 | 31.00 | 0.00 | 31.00 | 0.00 | 14.00 | 0.00 | 45.00 | 0.00 |
| E | I | Carnivora | FMNH 68725 | Hemigalus derbyanus boiei | Asia | 16.00 | 2.83 | 4.50 | 2.12 | 20.50 | 4.95 | 34.50 | 6.36 | 11.50 | 2.12 | 46.00 | 8.49 |
| E | C | Carnivora | FMNH 101928 | Hyaena hyaena dubbah | Africa | 22.25 | 4.43 | 5.75 | 3.51 | 28.00 | 2.31 | 18.00 | 4.24 | 5.75 | 1.50 | 23.75 | 4.03 |
| E | C | Carnivora | FMNH 101947 | Hyaena hyaena dubbah | Africa | 16.25 | 3.95 | 6.25 | 3.72 | 22.50 | 5.45 | 35.50 | 6.24 | 9.50 | 1.73 | 45.00 | 4.95 |
| E | C | Carnivora | FMNH 101982 | Hyaena hyaena dubbah | Africa | 16.25 | 3.30 | 3.75 | 2.25 | 20.00 | 2.45 | 37.00 | 4.40 | 24.25 | 5.06 | 61.25 | 3.30 |
| E | C | Carnivora | FMNH 101985 | Hyaena hyaena dubbah | Africa | 16.50 | 7.78 | 7.50 | 0.71 | 24.00 | 8.49 | 32.50 | 9.19 | 11.00 | 1.41 | 43.50 | 10.61 |
| E | C | Carnivora | FMNH 30778 | Panthera leo | Africa | 18.75 | 2.63 | 1.00 | 1.46 | 19.75 | 1.50 | 36.00 | 3.37 | 20.00 | 4.55 | 56.00 | 7.48 |
| E | C | Carnivora | FMNH 89926 | Panthera leo | Africa | 12.00 | 00.0 | 3.00 | 0.00 | 15.00 | 0.00 | 29.00 | 0.00 | 3.00 | 0.00 | 32.00 | 0.00 |
| E | C | Carnivora | FMNH 75609 | Panthera leo | Africa | 18.75 | 2.63 | 1.00 | 1.46 | 19.75 | 1.50 | 36.00 | 3.67 | 20.00 | 4.55 | 56.00 | 7.48 |
| E | C | Carnivora | FMNH 127842 | Panthera pardus | Africa | 17.75 | 3.30 | 3.00 | 1.00 | 20.75 | 2.99 | 41.50 | 2.08 | 21.00 | 8.41 | 62.50 | 10.34 |
| E | C | Carnivora | FMNH 30779 | Panthera pardus chui | Africa | 12.75 | 2.75 | 3.50 | 1.00 | 16.25 | 2.22 | 18.25 | 1.71 | 12.00 | 4.55 | 30.25 | 5.74 |
| E | C | Carnivora | FMNH 1446 | Panthera pardus nanopardus | Africa | 22.00 | 4.24 | 0.75 | 1.50 | 22.75 | 5.19 | 36.50 | 13.82 | 21.25 | 8.50 | 57.75 | 13.38 |
| E | C | Carnivora | FMNH 31152 | Panthera tigris tigris | Asia | 18.25 | 2.06 | 3.75 | 2.79 | 22.00 | 2.16 | 32.25 | 5.12 | 13.25 | 2.63 | 45.50 | 3.42 |
| E | C | Carnivora | FMNH 31153 | Panthera tigris tigris | Asia | 17.50 | 5.45 | 4.50 | 4.21 | 22.00 | 4.83 | 29.25 | 5.38 | 13.25 | 6.55 | 42.50 | 11.27 |
| M | B | Diprotodontida | AMNH 183408 | Aepyprymnus rufescens | Australasia | 25.33 | 4.04 | 2.33 | 2.15 | 27.67 | 4.73 | 31.00 | 2.00 | 12.00 | 3.61 | 43.00 | 2.65 |
| M | B | Diprotodontida | AMNH 18078 | Aepyprymnus rufescens | Australasia | 29.00 | 0.00 | 7.00 | 0.00 | 36.00 | 0.00 | 31.00 | 0.00 | 11.00 | 0.00 | 42.00 | 0.00 |
| M | B | Diprotodontida | AMNH 100981 | Ailurops ursinus furvus | Australasia | 24.00 | 0.00 | 0.00 | 0.00 | 24.00 | 0.00 | 26.00 | 0.00 | 14.00 | 0.00 | 40.00 | 0.00 |
| M | B | Diprotodontida | AMNH 153358 | Ailurops ursinus togianus | Australasia | 28.00 | 0.00 | 2.00 | 0.00 | 30.00 | 0.00 | 29.00 | 0.00 | 20.00 | 0.00 | 49.00 | 0.00 |
| M | I | Diprotodontida | AMNH 105943 | Dactylopsila trivirgata kataui | Australasia | 28.00 | 3.51 | 4.00 | 0.58 | 32.00 | 4.04 | 17.00 | 3.00 | 7.50 | 0.50 | 24.50 | 3.50 |
| M | I | Diprotodontida | AMNH 105941 | Dactylopsila trivirgata kataui | Australasia | 25.00 | 0.00 | 0.00 | 0.00 | 25.00 | 0.00 | 24.00 | 0.00 | 18.00 | 0.00 | 42.00 | 0.00 |
| M | C | Diprotodontida | AMNH 196843 | Dasyurus hallucatus | Australasia | 28.00 | 0.00 | 10.00 | 0.00 | 38.00 | 0.00 | 27.00 | 0.00 | 19.00 | 0.00 | 46.00 | 0.00 |
| M | C | Diprotodontida | AMNH 196846 | Dasyurus hallucatus | Australasia | 26.00 | 0.00 | 5.00 | 0.00 | 31.00 | 0.00 | 29.00 | 0.00 | 14.00 | 0.00 | 43.00 | 0.00 |
| M | B | Diprotodontida | AMNH 157282 | Dendrolagus dorianus dorianus | Australasia | 21.00 | 2.16 | 3.50 | 1.00 | 24.50 | 2.65 | 17.50 | 5.97 | 7.50 | 3.42 | 25.00 | 8.16 |
| M | B | Diprotodontida | AMNH 190997 | Dendrolagus dorianus notatus | Australasia | 25.00 | 0.00 | 4.00 | 0.00 | 29.00 | 0.00 | 19.00 | 0.00 | 13.00 | 0.00 | 32.00 | 0.00 |
| M | B | Diprotodontida | AMNH 198081 | Dendrolagus inustus finschi | Australasia | 23.00 | 6.24 | 1.67 | 2.10 | 24.67 | 8.08 | 17.00 | 5.57 | 10.67 | 6.43 | 27.67 | 11.59 |
| M | B | Diprotodontida | AMNH 65253 | Dendrolagus lumholtzi | Australasia | 22.75 | 0.96 | 0.25 | 0.50 | 23.00 | 0.82 | 18.00 | 10.13 | 6.00 | 1.83 | 24.00 | 8.98 |
| M | B | Diprotodontida | AMNH 65255 | Dendrolagus lumholtzi | Australasia | 20.00 | 2.94 | 1.00 | 2.00 | 21.00 | 4.83 | 15.50 | 2.38 | 5.00 | 2.45 | 20.50 | 4.12 |
| M | B | Diprotodontida | FMNH 60894 | Dendrolagus lumholtzi lumholtzi | Australasia | 14.50 | 1.29 | 0.25 | 0.50 | 14.75 | 1.50 | 20.75 | 2.36 | 11.00 | 2.45 | 31.75 | 4.65 |
| M | B | Diprotodontida | FMNH 60896 | Dendrolagus lumholtzi lumholtzi | Australasia | 13.25 | 2.63 | 0.50 | 0.58 | 13.75 | 3.20 | 17.75 | 2.63 | 11.25 | 4.03 | 29.00 | 5.48 |
| M | B | Diprotodontida | AMNH 109466 | Dorcopsis hageni | Australasia | 23.67 | 3.21 | 3.00 | 1.00 | 26.67 | 4.16 | 21.00 | 2.65 | 12.00 | 4.00 | 33.00 | 1.73 |
| M | G | Diprotodontida | AMNH 105990 | Dorcopsis muelleri veterum | Australasia | 25.50 | 4.95 | 2.50 | 2.12 | 28.00 | 7.07 | 26.00 | 1.41 | 10.00 | 2.83 | 36.00 | 1.41 |
| M | B | Diprotodontida | AMNH 154375 | Hemibelideus lemuroides | Australasia | 22.33 | 4.62 | 0.67 | 0.58 | 23.00 | 4.36 | 20.67 | 8.08 | 8.00 | 3.61 | 28.67 | 10.12 |
| M | B | Diprotodontida | AMNH 183365 | Hemibelideus lemuroides | Australasia | 19.00 | 0.00 | 0.00 | 0.00 | 19.00 | 0.00 | 23.00 | 0.00 | 8.00 | 0.00 | 31.00 | 0.00 |
| M | B | Diprotodontida | AMNH 183367 | Hemibelideus lemuroides | Australasia | 26.33 | 1.53 | 0.67 | 0.58 | 27.00 | 2.00 | 16.00 | 7.21 | 9.00 | 3.61 | 25.00 | 10.82 |
| M | I | Diprotodontida | AMNH 154496 | Hypsiprymnodon moschatus | Australasia | 23.00 | 0.00 | 5.00 | 0.00 | 28.00 | 0.00 | 22.00 | 0.00 | 11.00 | 0.00 | 33.00 | 0.00 |
| M | I | Diprotodontida | FMNH 60949 | Isoodon macrourus torosus | Australasia | 29.67 | 4.73 | 1.67 | 2.89 | 31.33 | 3.51 | 41.33 | 8.50 | 12.00 | 3.46 | 53.33 | 11.68 |
| M | G | Diprotodontida | FMNH 98913 | Macropus fuliginosus | Australasia | 32.50 | 4.36 | 7.75 | 2.67 | 40.25 | 4.19 | 25.25 | 3.30 | 12.00 | 2.71 | 37.25 | 5.44 |
| M | G | Diprotodontida | FMNH 119850 | Macropus fuliginosus | Australasia | 36.25 | 8.62 | 6.50 | 3.89 | 42.75 | 8.42 | 23.00 | 5.29 | 12.50 | 2.65 | 35.50 | 7.14 |
| M | G | Diprotodontida | AMNH 197018 | Macropus fuliginosus ocydromus | Australasia | 29.75 | 4.19 | 5.25 | 3.60 | 35.00 | 4.97 | 27.25 | 2.06 | 11.25 | 0.96 | 38.50 | 1.29 |
| M | G | Diprotodontida | FMNH 160059 | Macropus giganteus | Australasia | 23.50 | 2.65 | 4.00 | 4.09 | 27.50 | 4.80 | 19.25 | 6.18 | 10.00 | 4.55 | 29.25 | 8.42 |
| M | G | Diprotodontida | FMNH 160060 | Macropus giganteus | Australasia | 25.50 | 1.29 | 3.25 | 1.53 | 28.75 | 2.75 | 19.00 | 2.45 | 9.75 | 2.22 | 28.75 | 3.69 |
| M | G | Diprotodontida | FMNH 64427 | Macropus giganteus giganteus | Australasia | 31.50 | 5.20 | 6.50 | 3.50 | 38.00 | 4.69 | 20.25 | 0.96 | 14.25 | 1.71 | 34.50 | 1.00 |
| M | G | Diprotodontida | AMNH 65014 | Macropus giganteus giganteus | Australasia | 28.75 | 4.79 | 4.50 | 0.58 | 33.25 | 5.32 | 26.50 | 1.29 | 9.00 | 2.45 | 35.50 | 1.91 |
| M | G | Diprotodontida | FMNH 64355 | Macropus parryi | Australasia | 25.33 | 4.51 | 4.67 | 2.53 | 30.00 | 3.00 | 17.00 | 1.00 | 6.33 | 1.15 | 23.33 | 2.08 |
| M | G | Diprotodontida | FMNH 104670 | Macropus robustus | Australasia | 26.50 | 5.07 | 3.50 | 2.00 | 30.00 | 5.48 | 19.25 | 2.36 | 17.25 | 2.75 | 36.50 | 1.91 |
| M | G | Diprotodontida | FMNH 104674 | Macropus robustus | Australasia | 39.75 | 2.63 | 5.00 | 2.97 | 44.75 | 1.71 | 17.00 | 1.15 | 12.25 | 3.30 | 29.25 | 2.22 |
| M | G | Diprotodontida | FMNH 104678 | Macropus robustus | Australasia | 25.67 | 1.53 | 6.00 | 2.31 | 31.67 | 3.21 | 25.33 | 2.08 | 6.67 | 2.08 | 32.00 | 1.00 |
| M | G | Diprotodontida | FMNH 104685 | Macropus robustus | Australasia | 34.50 | 4.51 | 3.00 | 3.83 | 37.00 | 2.89 | 18.25 | 4.57 | 21.00 | 9.27 | 39.25 | 12.28 |
| M | G | Diprotodontida | FMNH 104688 | Macropus robustus | Australasia | 22.00 | 3.61 | 5.33 | 2.31 | 27.33 | 3.79 | 23.67 | 3.06 | 10.33 | 3.79 | 34.00 | 1.73 |
| M | G | Diprotodontida | FMNH 194689 | Macropus robustus | Australasia | 26.00 | 0.00 | 3.00 | 0.00 | 29.00 | 0.00 | 24.00 | 0.00 | 13.00 | 0.00 | 37.00 | 0.00 |
| M | G | Diprotodontida | FMNH 110828 | Macropus robustus | Australasia | 30.67 | 2.31 | 2.67 | 3.15 | 30.33 | 5.03 | 18.67 | 5.03 | 16.00 | 3.61 | 34.67 | 8.14 |
| M | G | Diprotodontida | AMNH 70183 | Macropus rufogriseus | Australasia | 30.33 | 3.21 | 6.33 | 1.58 | 36.67 | 4.62 | 27.67 | 5.03 | 9.00 | 3.61 | 36.67 | 5.69 |
| M | G | Diprotodontida | AMNH 69458 | Macropus rufogriseus | Australasia | 25.00 | 0.00 | 4.00 | 0.00 | 29.00 | 0.00 | 34.00 | 0.00 | 13.00 | 0.00 | 47.00 | 0.00 |
| M | G | Diprotodontida | FMNH 64611 | Macropus rufus | Australasia | 27.25 | 3.86 | 12.00 | 2.98 | 39.25 | 5.91 | 18.25 | 2.22 | 9.25 | 2.22 | 27.50 | 3.42 |
| M | G | Diprotodontida | AMNH 196999 | Macropus rufus | Australasia | 29.00 | 1.00 | 5.50 | 2.50 | 34.50 | 4.95 | 18.00 | 4.24 | 3.50 | 0.71 | 21.50 | 3.54 |
| M | I | Diprotodontida | AMNH 160202 | Perameles nasuta | Australasia | 31.00 | 0.00 | 0.00 | 0.00 | 31.00 | 0.00 | 20.00 | 0.00 | 6.00 | 0.00 | 26.00 | 0.00 |
| M | I | Diprotodontida | AMNH 160418 | Perameles nasuta | Australasia | 21.00 | 0.00 | 3.00 | 0.00 | 24.00 | 0.00 | 22.00 | 0.00 | 9.00 | 0.00 | 31.00 | 0.00 |
| M | I | Diprotodontida | AMNH 160208 | Perameles nasuta nasuta | Australasia | 36.00 | 0.00 | 1.00 | 0.00 | 36.00 | 2.35 | 28.00 | 3.11 | 8.00 | 2.16 | 36.00 | 1.29 |
| M | I | Diprotodontida | FMNH 60942 | Perameles nasuta pallescens | Australasia | 35.00 | 0.00 | 7.00 | 0.00 | 42.00 | 0.00 | 26.00 | 0.00 | 28.00 | 0.00 | 54.00 | 0.00 |
| M | B | Diprotodontida | FMNH 64331 | Petauroides volans minor | Australasia | 21.00 | 0.00 | 0.00 | 0.00 | 21.00 | 0.00 | 19.00 | 0.00 | 14.00 | 0.00 | 33.00 | 4.95 |
| M | B | Diprotodontida | AMNH 107257 | Petauroides volans minor | Australasia | 30.50 | 4.95 | 1.00 | 0.00 | 31.50 | 4.95 | 14.50 | 6.36 | 12.00 | 0.00 | 26.50 | 6.36 |
| M | B | Diprotodontida | AMNH 107258 | Petauroides volans minor | Australasia | 23.00 | 5.66 | 3.50 | 4.95 | 26.50 | 0.71 | 16.00 | 4.24 | 8.50 | 3.54 | 24.50 | 7.78 |
| M | B | Diprotodontida | AMNH 63569 | Petauroides volans volans | Australasia | 20.00 | 0.00 | 2.00 | 0.00 | 22.00 | 0.00 | 22.00 | 0.00 | 7.00 | 0.00 | 29.00 | 0.00 |
| M | B | Diprotodontida | AMNH 257615 | Petauroides volans volans | Australasia | 24.00 | 2.83 | 3.00 | 1.41 | 27.00 | 4.24 | 21.50 | 7.78 | 9.00 | 2.83 | 30.50 | 4.95 |
| M | B | Diprotodontida | AMNH 257615 | Petauroides volans volans | Australasia | 18.00 | 0.00 | 0.00 | 0.00 | 18.00 | 0.00 | 8.00 | 0.00 | 12.00 | 0.00 | 20.00 | 0.00 |
| M | I | Diprotodontida | AMNH 107265 | Petaurus australis reginae | Australasia | 23.00 | 0.00 | 6.00 | 0.00 | 29.00 | 0.00 | 21.00 | 0.00 | 13.00 | 0.00 | 34.00 | 0.00 |
| M | I | Diprotodontida | AMNH 107266 | Petaurus australis reginae | Australasia | 25.00 | 0.00 | 2.00 | 0.00 | 27.00 | 0.00 | 29.00 | 0.00 | 11.00 | 0.00 | 40.00 | 0.00 |
| M | G | Diprotodontida | AMNH 107703 | Petrogale lateralis | Australasia | 30.75 | 4.99 | 4.25 | 2.52 | 35.00 | 4.90 | 21.50 | 3.51 | 10.75 | 2.22 | 32.25 | 4.99 |
| M | G | Diprotodontida | AMNH 107719 | Petrogale lateralis | Australasia | 20.00 | 0.00 | 8.00 | 0.00 | 28.00 | 0.00 | 26.00 | 0.00 | 10.00 | 0.00 | 36.00 | 0.00 |
| M | G | Diprotodontida | AMNH 107717 | Petrogale lateralis | Australasia | 33.50 | 2.12 | 5.00 | 0.00 | 38.50 | 2.12 | 16.50 | 6.36 | 11.00 | 2.83 | 27.50 | 9.19 |
| M | B | Diprotodontida | FMNH 81524 | Phascolarctos cinereus | Australasia | 17.00 | 2.16 | 0.25 | 0.50 | 17.25 | 2.06 | 13.00 | 2.45 | 11.75 | 3.30 | 24.75 | 1.89 |
| M | B | Diprotodontida | FMNH 112541 | Phascolarctos cinereus | Australasia | 18.25 | 0.96 | 0.50 | 0.58 | 18.75 | 0.50 | 10.50 | 2.08 | 8.25 | 2.06 | 18.75 | 2.63 |
| M | B | Diprotodontida | FMNH 112543 | Phascolarctos cinereus | Australasia | 14.50 | 2.38 | 1.25 | 1.50 | 15.75 | 3.40 | 14.25 | 2.22 | 12.75 | 2.99 | 27.00 | 3.56 |
| M | B | Diprotodontida | AMNH 90284 | Phascolarctos cinerus | Australasia | 24.33 | 2.31 | 0.33 | 0.58 | 24.67 | 2.08 | 16.33 | 4.51 | 9.33 | 4.04 | 25.67 | 2.52 |
| M | I | Diprotodontida | AMNH 65321 | Potorous tridactylus apicalis | Australasia | 30.00 | 0.00 | 10.00 | 0.00 | 40.00 | 0.00 | 22.00 | 0.00 | 21.00 | 0.00 | 43.00 | 0.00 |
| M | I | Diprotodontida | AMNH 65331 | Potorous tridactylus apicalis | Australasia | 27.00 | 0.00 | 7.00 | 0.00 | 34.00 | 0.00 | 21.00 | 0.00 | 17.00 | 0.00 | 38.00 | 0.00 |
| M | I | Diprotodontida | AMNH 65335 | Potorous tridactylus apicalis | Australasia | 32.00 | 0.00 | 7.00 | 0.00 | 39.00 | 0.00 | 35.00 | 0.00 | 19.00 | 0.00 | 54.00 | 0.00 |
| M | B | Diprotodontida | FMNH 60916 | Pseudocheirus archeri | Australasia | 17.50 | 0.71 | 3.00 | 1.41 | 20.50 | 0.71 | 12.00 | 2.83 | 12.50 | 4.95 | 24.50 | 7.78 |
| M | B | Diprotodontida | FMNH 60920 | Pseudocheirus archeri | Australasia | 18.50 | 3.54 | 0.50 | 0.71 | 19.00 | 2.83 | 18.50 | 2.12 | 21.00 | 2.83 | 39.50 | 4.95 |
| M | B | Diprotodontida | AMNH 151759 | Pseudocheirus mayeri | Australasia | 25.00 | 0.00 | 4.00 | 0.00 | 29.00 | 0.00 | 29.00 | 0.00 | 14.00 | 0.00 | 43.00 | 0.00 |
| M | B | Diprotodontida | AMNH 151643 | Pseudocheirus mayeri | Australasia | 25.00 | 0.00 | 2.00 | 0.00 | 27.00 | 0.00 | 23.00 | 0.00 | 12.00 | 0.00 | 35.00 | 0.00 |
| M | B | Diprotodontida | AMNH 108574 | Pseudochirops cupreus | Australasia | 19.00 | 0.00 | 0.00 | 0.00 | 19.00 | 0.00 | 28.00 | 0.00 | 8.00 | 0.00 | 36.00 | 0.00 |
| M | B | Diprotodontida | FMNH 67712 | Setonix brachyurus | Australasia | 13.00 | 0.00 | 0.00 | 0.00 | 13.00 | 0.00 | 26.00 | 0.00 | 18.00 | 0.00 | 38.00 | 0.00 |
| M | B | Diprotodontida | AMNH 74488 | Setonix brachyurus | Australasia | 15.00 | 0.00 | 5.00 | 0.00 | 20.00 | 0.00 | 27.00 | 0.00 | 19.00 | 0.00 | 46.00 | 0.00 |
| M | B | Diprotodontida | AMNH 197118 | Setonix brachyurus | Australasia | 25.00 | 0.00 | 6.00 | 0.00 | 31.00 | 0.00 | 25.00 | 0.00 | 20.00 | 0.00 | 45.00 | 0.00 |
| M | B | Diprotodontida | AMNH 160254 | Setonix brachyurus | Australasia | 21.75 | 2.99 | 3.00 | 2.21 | 24.75 | 3.10 | 19.25 | 2.22 | 13.50 | 3.32 | 32.75 | 5.12 |
| M | B | Diprotodontida | AMNH 74491 | Setonix brachyurus | Australasia | 31.00 | 1.41 | 4.50 | 2.12 | 35.50 | 3.54 | 25.50 | 5.07 | 15.00 | 3.40 | 40.50 | 3.54 |
| M | B | Diprotodontida | AMNH 157174 | Spilocuscus maculatus | Australasia | 26.67 | 4.73 | 7.00 | 3.46 | 33.67 | 3.51 | 24.33 | 4.93 | 13.67 | 4.73 | 38.00 | 9.64 |
| M | B | Diprotodontida | AMNH 198110 | Spilocuscus maculatus maculatus | Australasia | 16.50 | 3.54 | 0.50 | 0.71 | 17.00 | 4.24 | 17.00 | 1.41 | 8.50 | 0.71 | 25.50 | 2.12 |
| M | B | Diprotodontida | AMNH 153697 | Spilocuscus maculatus nudicaudatus | Australasia | 28.00 | 1.41 | 3.50 | 2.12 | 31.50 | 3.54 | 26.50 | 3.54 | 12.50 | 0.71 | 39.00 | 2.83 |
| M | B | Diprotodontida | AMNH 151821 | Spilocuscus maculatus nudicaudatus | Australasia | 25.67 | 1.53 | 0.67 | 1.15 | 26.33 | 0.58 | 25.67 | 6.66 | 8.67 | 0.58 | 34.33 | 6.81 |
| M | C | Diprotodontida | FMNH 81522 | Thylacinus cynocephalus | Australasia | 17.50 | 5.26 | 7.25 | 2.33 | 24.75 | 5.19 | 21.50 | 4.36 | 21.25 | 5.68 | 42.75 | 9.57 |
| M | G | Diprotodontida | AMNH 65208 | Thylogale billiardii | Australasia | 19.00 | 0.00 | 3.00 | 0.00 | 22.00 | 0.00 | 18.00 | 0.00 | 10.00 | 0.00 | 28.00 | 0.00 |
| M | B | Diprotodontida | AMNH 160239 | Trichosurus caninus caninus | Australasia | 26.33 | 2.52 | 5.33 | 1.58 | 31.67 | 3.51 | 26.33 | 5.51 | 12.67 | 4.04 | 39.00 | 7.00 |
| M | B | Diprotodontida | AMNH 160240 | Trichosurus caninus caninus | Australasia | 28.00 | 2.65 | 3.67 | 1.53 | 31.67 | 1.53 | 21.33 | 1.53 | 13.67 | 3.51 | 35.00 | 4.58 |
| M | B | Diprotodontida | FMNH 143375 | Trichosurus vulpecula | Australasia | 15.50 | 3.11 | 0.00 | 0.00 | 15.50 | 3.11 | 19.25 | 2.22 | 15.50 | 2.89 | 34.75 | 2.22 |
| M | B | Diprotodontida | FMNH 57800 | Trichosurus vulpecula fuliginosus | Australasia | 17.75 | 4.35 | 0.25 | 0.50 | 18.00 | 4.08 | 17.00 | 4.32 | 12.50 | 1.91 | 29.50 | 5.97 |
| M | G | Diprotodontida | FMNH 98887 | Vombatus ursinus tasmaniensis | Australasia | 17.00 | 3.00 | 7.33 | 3.22 | 24.33 | 3.21 | 13.67 | 1.53 | 11.67 | 0.58 | 25.33 | 2.08 |
| E | I | Erinaceae | FMNH 96339 | Erinaceus concolor | Asia | 17.00 | 2.83 | 8.50 | 2.12 | 25.50 | 2.12 | 24.50 | 9.19 | 14.00 | 4.24 | 38.50 | 4.95 |
| E | I | Erinaceae | FMNH 96340 | Erinaceus concolor | Asia | 18.00 | 0.00 | 3.00 | 0.00 | 21.00 | 0.00 | 37.00 | 0.00 | 14.00 | 0.00 | 51.00 | 0.00 |
| E | I | Erinaceae | FMNH 29723 | Hemiechinus auritus turfanicus | Asia | 21.50 | 0.71 | 10.50 | 4.95 | 32.00 | 1.41 | 24.50 | 2.12 | 15.50 | 2.12 | 40.00 | 0.00 |
| E | G | Perissodactyla | FMNH 125413 | Ceratotherium simum simum | Africa | 19.00 | 2.58 | 10.75 | 4.83 | 29.75 | 4.03 | 18.00 | 1.41 | 8.25 | 4.03 | 26.25 | 4.19 |
| E | B | Perissodactyla | FMNH 85429 | Diceros bicornis | Africa | 18.00 | 3.46 | 3.00 | 1.46 | 21.00 | 3.74 | 19.50 | 2.52 | 11.25 | 0.96 | 30.75 | 2.50 |
| E | G | Perissodactyla | FMNH 129753 | Equus caballus | Asia | 29.33 | 6.11 | 5.33 | 2.58 | 34.67 | 7.23 | 12.33 | 3.21 | 9.33 | 2.08 | 21.67 | 5.03 |
| E | G | Perissodactyla | FMNH 41095 | Equus caballus | North America | 17.00 | 2.83 | 10.50 | 3.54 | 27.50 | 0.71 | 18.50 | 6.36 | 11.50 | 3.54 | 30.00 | 9.90 |
| E | G | Perissodactyla | FMNH 135203 | Equus caballus | North America | 18.00 | 5.35 | 8.25 | 2.23 | 26.25 | 4.43 | 18.00 | 4.76 | 11.00 | 0.82 | 29.00 | 4.24 |
| E | G | Perissodactyla | FMNH 92902 | Equus caballus caballus | Asia | 14.25 | 1.71 | 8.75 | 2.55 | 23.00 | 2.31 | 24.00 | 6.48 | 11.25 | 2.63 | 35.25 | 6.24 |
| E | G | Perissodactyla | FMNH 97880 | Equus hemionus | Asia | 30.50 | 7.78 | 9.50 | 2.12 | 40.00 | 5.66 | 14.50 | 0.71 | 9.50 | 6.36 | 24.00 | 5.66 |
| E | H | Perissodactyla | FMNH 15979 | Tapirus bairdii | North America | 20.75 | 2.75 | 8.75 | 3.53 | 29.50 | 1.91 | 35.50 | 6.03 | 26.25 | 4.11 | 61.75 | 8.14 |
| E | H | Perissodactyla | FMNH 69550 | Tapirus bairdii | North America | 19.25 | 3.40 | 7.25 | 3.65 | 26.50 | 2.89 | 36.00 | 4.97 | 24.50 | 7.59 | 60.50 | 10.25 |
| E | H | Perissodactyla | FMNH 66017 | Tapirus indicus | Asia | 13.25 | 1.71 | 11.50 | 6.86 | 24.75 | 6.45 | 35.25 | 10.66 | 36.25 | 2.22 | 71.50 | 12.15 |
| E | H | Primates | FMNH 68858 | Aotus lemurinus griseimembra | South America | 11.00 | 0.00 | 6.00 | 0.00 | 17.00 | 4.45 | 39.00 | 0.00 | 26.00 | 0.00 | 65.00 | 19.86 |
| E | H | Primates | FMNH 88250 | Cacajao melanocephalus ouakary | South America | 12.00 | 0.00 | 7.00 | 0.00 | 19.00 | 0.00 | 39.00 | 0.00 | 18.00 | 0.00 | 57.00 | 0.00 |
| E | H | Primates | FMNH 20001 | Chiropotes satanas chiropotes | South America | 23.00 | 0.00 | 9.00 | 0.00 | 32.00 | 0.00 | 36.00 | 0.00 | 21.00 | 0.00 | 57.00 | 0.00 |
| E | B | Primates | FMNH 31163 | Colobus guereza kikuyuensis | Africa | 13.25 | 1.89 | 1.00 | 1.41 | 14.25 | 1.71 | 17.75 | 1.71 | 13.25 | 6.90 | 31.00 | 7.39 |
| E | B | Primates | FMNH 35117 | Colobus guereza kikuyuensis | Africa | 11.00 | 2.65 | 1.67 | 2.89 | 12.67 | 0.58 | 14.00 | 2.65 | 18.33 | 4.51 | 32.33 | 2.08 |
| E | B | Primates | FMNH 43107 | Nasalis larvatus | Asia | 11.50 | 2.12 | 0.50 | 0.71 | 12.00 | 1.41 | 23.50 | 0.71 | 6.00 | 4.24 | 29.50 | 4.95 |
| E | B | Primates | FMNH 68682 | Nasalis larvatus | Asia | 10.50 | 0.71 | 1.00 | 1.41 | 11.50 | 2.12 | 22.50 | 4.95 | 19.00 | 5.66 | 41.50 | 10.61 |
| E | H | Primates | FMNH 27553 | Pan troglodytes | Africa | 27.00 | 2.94 | 4.25 | 1.53 | 31.25 | 2.63 | 30.75 | 5.25 | 22.00 | 2.83 | 52.75 | 4.11 |
| E | H | Primates | FMNH 93253 | Pithecia pithecia pithecia | South America | 17.00 | 0.00 | 5.00 | 0.00 | 22.00 | 0.00 | 38.00 | 0.00 | 14.00 | 0.00 | 52.00 | 0.00 |
| E | H | Primates | FMNH 95504 | Pithecia pithecia pithecia | South America | 19.00 | 0.00 | 5.00 | 0.00 | 24.00 | 0.00 | 40.00 | 0.00 | 16.00 | 0.00 | 56.00 | 0.00 |
| E | G | Primates | FMNH 27185 | Theropithecus gelada | Africa | 34.33 | 4.16 | 3.33 | 3.26 | 37.67 | 4.04 | 27.33 | 3.21 | 11.33 | 1.53 | 38.67 | 3.06 |
| E | G | Primates | FMNH 27186 | Theropithecus gelada | Africa | 30.33 | 6.35 | 5.67 | 5.16 | 36.00 | 9.64 | 20.33 | 2.08 | 11.33 | 3.21 | 31.67 | 4.04 |
| E | H | Rodentia | FMNH 43531 | Callosciurus prevostii atricapillus | Asia | 27.00 | 0.00 | 9.00 | 0.00 | 36.00 | 0.00 | 39.00 | 0.00 | 12.00 | 0.00 | 51.00 | 0.00 |
| E | H | Rodentia | FMNH 43532 | Callosciurus prevostii atricapillus | Asia | 26.00 | 0.00 | 6.00 | 0.00 | 32.00 | 0.00 | 42.00 | 0.00 | 22.00 | 0.00 | 64.00 | 0.00 |
| E | H | Rodentia | FMNH 141040 | Callosciurus prevostii caedis | Asia | 30.00 | 0.00 | 6.00 | 0.00 | 36.00 | 0.00 | 39.00 | 0.00 | 12.00 | 0.00 | 51.00 | 0.00 |
| E | H | Rodentia | FMNH 38827 | Gerbilliscus leucogaster bechuanae | Africa | 20.00 | 0.00 | 3.00 | 0.00 | 23.00 | 0.00 | 21.00 | 0.00 | 15.00 | 0.00 | 36.00 | 0.00 |
| E | H | Rodentia | FMNH 38828 | Gerbilliscus leucogaster bechuanae | Africa | 26.00 | 0.00 | 3.00 | 0.00 | 29.00 | 0.00 | 20.00 | 0.00 | 16.00 | 0.00 | 36.00 | 0.00 |
| E | H | Rodentia | FMNH 77593 | Gerbilliscus leucogaster schinzi | Africa | 23.00 | 0.00 | 4.00 | 0.00 | 27.00 | 0.00 | 24.00 | 0.00 | 8.00 | 0.00 | 32.00 | 0.00 |
| E | H | Rodentia | FMNH 204179 | Mus bufo | Africa | 30.00 | 0.00 | 10.00 | 0.00 | 40.00 | 0.00 | 31.00 | 0.00 | 18.00 | 0.00 | 49.00 | 0.00 |
| E | H | Rodentia | FMNH 42043 | Ototylomys phyllotis connectens | North America | 31.00 | 0.00 | 7.00 | 0.00 | 38.00 | 0.00 | 48.00 | 0.00 | 24.00 | 0.00 | 72.00 | 0.00 |
| E | H | Rodentia | FMNH 64564 | Ototylomys phyllotis connectens | North America | 31.00 | 0.00 | 6.00 | 0.00 | 37.00 | 0.00 | 36.00 | 0.00 | 16.00 | 0.00 | 52.00 | 0.00 |
| E | H | Rodentia | FMNH 64565 | Ototylomys phyllotis connectens | North America | 28.00 | 0.00 | 7.00 | 0.00 | 35.00 | 0.00 | 43.00 | 0.00 | 27.00 | 0.00 | 70.00 | 0.00 |
| E | H | Rodentia | FMNH 93065 | Oxymycterus sp. | South America | 25.00 | 0.00 | 8.00 | 0.00 | 33.00 | 0.00 | 30.00 | 0.00 | 21.00 | 0.00 | 51.00 | 0.00 |
| E | H | Rodentia | FMNH 35251 | Oxymycterus paramensis jacentior | South America | 34.00 | 0.00 | 7.00 | 0.00 | 41.00 | 0.00 | 41.00 | 0.00 | 13.00 | 0.00 | 54.00 | 0.00 |
| E | H | Rodentia | FMNH 125307 | Peromyscus leucopus tornillo | North America | 29.00 | 0.00 | 6.00 | 0.00 | 35.00 | 0.00 | 46.00 | 0.00 | 14.00 | 0.00 | 60.00 | 0.00 |
| E | H | Rodentia | FMNH 35431 | Ratufa bicolor gigantea | Asia | 26.00 | 0.00 | 6.00 | 0.00 | 32.00 | 0.00 | 46.00 | 0.00 | 20.00 | 0.00 | 66.00 | 0.00 |
| E | H | Rodentia | FMNH 35432 | Ratufa bicolor gigantea | Asia | 23.50 | 0.71 | 5.50 | 3.54 | 29.00 | 2.83 | 38.00 | 0.00 | 15.00 | 1.41 | 53.00 | 1.41 |
| E | H | Rodentia | FMNH 84846 | Ratufa bicolor gigantea | Asia | 25.50 | 2.12 | 6.00 | 4.24 | 31.50 | 6.36 | 29.50 | 10.61 | 9.50 | 0.71 | 39.00 | 11.31 |
| E | H | Rodentia | FMNH 84847 | Ratufa bicolor gigantea | Asia | 19.00 | 0.00 | 3.00 | 0.00 | 22.00 | 0.00 | 40.00 | 0.00 | 15.00 | 0.00 | 55.00 | 0.00 |
| E | H | Rodentia | FMNH 90786 | Sciurus carolinensis pennsylvanicus | North America | 10.33 | 1.15 | 4.33 | 4.06 | 14.67 | 4.93 | 34.67 | 6.66 | 33.67 | 1.53 | 68.33 | 8.08 |
| E | H | Rodentia | FMNH 71216 | Tylomys mirae | South America | 29.00 | 0.00 | 4.00 | 0.00 | 33.00 | 0.00 | 44.00 | 0.00 | 15.00 | 0.00 | 59.00 | 0.00 |
| E | H | Rodentia | FMNH 71217 | Tylomys mirae | South America | 20.00 | 0.00 | 10.00 | 0.00 | 30.00 | 0.00 | 46.00 | 0.00 | 19.00 | 0.00 | 65.00 | 0.00 |
| E | G | Rodentia | FMNH 69127 | Zygodontomys brevicauda cherriei | South America | 36.00 | 0.00 | 11.00 | 0.00 | 47.00 | 0.00 | 28.00 | 0.00 | 11.00 | 0.00 | 39.00 | 0.00 |
| E | I | Soricomorpha | FMNH 199561 | Blarina brevicauda talpoides | North America | 29.00 | 0.00 | 10.00 | 0.00 | 39.00 | 0.00 | 36.00 | 0.00 | 24.00 | 0.00 | 60.00 | 0.00 |
| E | I | Soricomorpha | FMNH 35858 | Nectogale elegans sikhimensis | Asia | 13.00 | 0.00 | 19.00 | 0.00 | 32.00 | 0.00 | 40.00 | 0.00 | 29.00 | 0.00 | 69.00 | 0.00 |
| E | I | Soricomorpha | FMNH 35859 | Nectogale elegans sikhimensis | Asia | 17.00 | 0.00 | 5.00 | 0.00 | 22.00 | 0.00 | 47.00 | 0.00 | 16.00 | 0.00 | 63.00 | 0.00 |
| E | I | Soricomorpha | FMNH 35860 | Nectogale elegans sikhimensis | Asia | 20.00 | 0.00 | 16.00 | 0.00 | 36.00 | 0.00 | 51.00 | 0.00 | 20.00 | 0.00 | 71.00 | 0.00 |
| E | I | Soricomorpha | FMNH 65353 | Neomys fodiens fodiens | Europe | 20.00 | 0.00 | 3.00 | 0.00 | 23.00 | 0.00 | 37.00 | 0.00 | 10.00 | 0.00 | 47.00 | 0.00 |
| E | I | Soricomorpha | FMNH 66264 | Neomys fodiens fodiens | Europe | 25.00 | 0.00 | 4.00 | 0.00 | 29.00 | 0.00 | 29.00 | 0.00 | 11.00 | 0.00 | 40.00 | 0.00 |
| E | I | Soricomorpha | FMNH 66265 | Neomys fodiens fodiens | Europe | 20.00 | 0.00 | 4.00 | 0.00 | 24.00 | 0.00 | 30.00 | 0.00 | 11.00 | 0.00 | 41.00 | 0.00 |
| E | I | Soricomorpha | FMNH 9743 | Neurotrichus gibbsii gibbsii | North America | 23.00 | 0.00 | 7.00 | 0.00 | 30.00 | 0.00 | 30.00 | 0.00 | 10.00 | 0.00 | 40.00 | 0.00 |
| E | I | Soricomorpha | FMNH 8154 | Scalopus aquaticus machrinus | North America | 32.00 | 0.00 | 3.00 | 0.00 | 35.00 | 0.00 | 48.00 | 0.00 | 13.00 | 0.00 | 61.00 | 0.00 |
| E | I | Soricomorpha | FMNH 148269 | Scutisorex somereni | Africa | 25.00 | 0.00 | 2.00 | 0.00 | 27.00 | 0.00 | 36.00 | 0.00 | 18.00 | 0.00 | 54.00 | 0.00 |
| E | I | Soricomorpha | FMNH 148941 | Scutisorex somereni | Africa | 29.00 | 0.00 | 1.00 | 0.00 | 30.00 | 0.00 | 43.00 | 0.00 | 9.00 | 0.00 | 52.00 | 0.00 |
| E | I | Soricomorpha | FMNH 134 | Solenodon cubanus | North America | 25.00 | 0.00 | 15.00 | 0.00 | 40.00 | 0.00 | 48.00 | 0.00 | 15.00 | 0.00 | 63.00 | 0.00 |
| E | I | Soricomorpha | FMNH 18505 | Solenodon paradoxus | North America | 36.00 | 0.00 | 12.00 | 0.00 | 48.00 | 0.00 | 40.00 | 0.00 | 22.00 | 0.00 | 62.00 | 0.00 |
| E | I | Soricomorpha | FMNH 104110 | Soriculus nigrescens nigrescens | Asia | 29.00 | 0.00 | 3.00 | 0.00 | 32.00 | 0.00 | 34.00 | 0.00 | 13.00 | 0.00 | 47.00 | 0.00 |
| E | I | Soricomorpha | FMNH 104115 | Soriculus nigrescens nigrescens | Asia | 30.00 | 0.00 | 2.00 | 0.00 | 32.00 | 0.00 | 29.00 | 0.00 | 10.00 | 0.00 | 39.00 | 0.00 |
| E | I | Soricomorpha | FMNH 34797 | Suncus murinus caerulescens | Asia | 27.00 | 0.00 | 2.00 | 0.00 | 29.00 | 0.00 | 32.00 | 0.00 | 13.00 | 0.00 | 45.00 | 0.00 |
| E | I | Soricomorpha | FMNH 151141 | Sylvisorex howelli | Africa | 24.00 | 0.00 | 5.00 | 0.00 | 29.00 | 0.00 | 17.00 | 0.00 | 6.00 | 0.00 | 23.00 | 0.00 |

**SECTION 2**

*Table S2*. Taxon list and species average measurements for Shearing Crest Score (SCS) and body mass. Number of individuals refers to the number of specimens included in the species average. Under Phylogeny, E= Eutherian, M=Metatherian; Diet Category, I=Insectivore, C=Carnivore, G=Grazer, B=Browser, H=Hard-Object Feeder.

| Eutherian/ Metatherian | Genus | Species | Total shear length (mm) | Molar Area (mm^2^) | SCS (mm) | Diet Category | Number of Individuals | Body Mass (kg) |
| --- | --- | --- | --- | --- | --- | --- | --- | --- |
| E | Echinops | telfairi | 7.60 | 6.62 | 2.94 | I | 5 | 0.13 |
| E | Geogale | aurita | 3.56 | 1.15 | 3.32 | I | 1 | 0.07 |
| E | Micropotamogale | lamottei | 5.67 | 4.57 | 2.65 | I | 1 | 0.07 |
| E | Potamogale | velox | 10.49 | 14.07 | 2.80 | I | 1 | 0.66 |
| E | Tenrec | ecaudatus | 9.19 | 8.98 | 3.07 | I | 4 | 0.87 |
| E | Bison | bison | 104.62 | 843.67 | 3.60 | G | 2 | 579.26 |
| E | Capra | hircus | 29.39 | 162.60 | 2.30 | B | 1 | 33.50 |
| E | Cephalophus | natalensi | 25.15 | 82.04 | 2.78 | B | 1 | 12.00 |
| E | Connochaetes | taurinus | 150.19 | 2521.08 | 2.99 | G | 1 | 179.90 |
| E | Kobus | ellipsiprymnus | 78.31 | 354.39 | 4.16 | G | 2 | 210.00 |
| E | Kobus | leche | 50.36 | 165.96 | 3.91 | B | 1 | 110.79 |
| E | Ovis | aries | 30.30 | 116.11 | 2.81 | G | 1 | 50.00 |
| E | Syncerus | caffer | 90.55 | 715.50 | 3.38 | G | 2 | 580.00 |
| E | Alces | alces | 80.11 | 668.95 | 3.10 | B | 1 | 355.00 |
| E | Blastocerus | dichotomus | 50.89 | 234.80 | 3.32 | G | 1 | 86.66 |
| E | Capreolus | capreolus | 46.68 | 160.82 | 3.68 | B | 1 | 22.50 |
| E | Odocoileus | hemionus | 55.06 | 206.92 | 3.83 | B | 1 | 54.20 |
| E | Odocoileus | virginianus | 35.00 | 171.80 | 2.67 | B | 1 | 55.50 |
| E | Rangifer | tarandus | 69.10 | 286.78 | 4.08 | B | 1 | 86.03 |
| E | Giraffa | camelopardis | 98.63 | 1071.08 | 3.03 | B | 3 | 899.99 |
| E | Okapia | johnstoni | 69.41 | 520.66 | 3.05 | B | 3 | 230.00 |
| E | Hippopotamus | amphibius | 72.29 | 1016.39 | 2.27 | G | 1 | 1417.49 |
| E | Tragulus | javanicus | 17.92 | 26.90 | 3.45 | H | 2 | 3.30 |
| E | Tragulus | napu | 18.71 | 27.59 | 3.56 | H | 1 | 5.90 |
| E | Felis | silvestris | 10.16 | 56.16 | 1.37 | C | 5 | 4.65 |
| E | Lynx | rufus | 13.75 | 105.06 | 1.35 | C | 4 | 8.90 |
| E | Panthera | tigris | 32.88 | 539.01 | 1.46 | C | 4 | 162.56 |
| E | Panthera | leo | 44.35 | 750.87 | 1.62 | C | 4 | 161.50 |
| E | Panthera | pardus | 18.93 | 184.55 | 1.39 | C | 4 | 55.00 |
| E | Crocuta | crocuta | 39.00 | 524.84 | 1.73 | C | 3 | 63.00 |
| E | Hyaena | hyaena | 33.66 | 640.25 | 1.33 | C | 2 | 46.00 |
| E | Ailuropoda | melanoleuca | 49.39 | 368.53 | 2.57 | G | 2 | 108.40 |
| E | Chrotogale | owstoni | 6.22 | 30.35 | 1.13 | I | 1 | 3.25 |
| E | Cynogale | bennettii | 6.35 | 48.52 | 0.91 | I | 1 | 4.50 |
| E | Hemigalus | derbyanus | 7.06 | 45.17 | 1.05 | I | 4 | 2.00 |
| M | Dasyurus | hallucatus | 9.96 | 11.28 | 2.96 | I | 1 | 0.53 |
| M | Myoictis | melas | 10.10 | 9.73 | 3.24 | I | 1 | 0.20 |
| M | Dendrolagus | dorianus | 16.27 | 41.26 | 2.53 | B | 2 | 9.58 |
| M | Dendrolagus | inustus | 20.59 | 25.40 | 4.09 | B | 1 | 12.63 |
| M | Dendrolagus | lumholtzi | 15.67 | 24.01 | 3.25 | B | 9 | 6.75 |
| M | Dorcopsis | hageni | 13.37 | 29.99 | 2.43 | G | 3 | 5.50 |
| M | Macropus | agilis | 14.27 | 47.52 | 2.08 | G | 4 | 15.00 |
| M | Macropus | antilopinus | 18.63 | 75.83 | 2.20 | G | 3 | 27.25 |
| M | Macropus | fuliginosus | 21.88 | 91.67 | 2.30 | G | 4 | 22.00 |
| M | Macropus | giganteus | 20.01 | 73.94 | 2.33 | G | 5 | 25.88 |
| M | Macropus | parryi | 14.21 | 49.55 | 2.02 | G | 2 | 13.50 |
| M | Macropus | rufrogresius | 11.75 | 31.22 | 2.11 | G | 2 | 16.85 |
| M | Macropus | rufus | 20.20 | 80.50 | 2.29 | G | 4 | 46.25 |
| M | Macropus | robustus | 19.38 | 81.74 | 2.17 | G | 12 | 21.26 |
| M | Onychogalea | unguifera | 12.20 | 26.21 | 2.38 | G | 1 | 6.65 |
| M | Thylogale | billiardii | 10.84 | 30.45 | 1.96 | G | 1 | 5.45 |
| M | Thylogale | stigmatica | 11.38 | 22.30 | 2.41 | G | 4 | 4.60 |
| M | Thylogale | thetis | 17.74 | 25.44 | 3.52 | G | 1 | 5.40 |
| M | Wallabia | bicolor | 14.07 | 35.76 | 2.40 | B | 2 | 15.00 |
| M | Hypsiprymnodon | moschatus | 9.75 | 6.31 | 3.88 | I | 1 | 0.52 |
| M | Isoodon | macrourus | 17.82 | 17.16 | 4.29 | I | 2 | 1.60 |
| M | Perameles | nasuta | 12.88 | 13.59 | 3.51 | I | 3 | 0.98 |
| M | Dactylopsila | palpator | 6.63 | 12.34 | 1.89 | I | 1 | 0.40 |
| M | Dactylopsila | trivirgata | 9.78 | 10.68 | 2.99 | I | 2 | 0.42 |
| M | Petaurus | australis | 7.63 | 6.08 | 3.10 | I | 3 | 0.58 |
| M | Petaurus | breviceps | 5.91 | 2.71 | 3.60 | I | 2 | 0.13 |
| M | Ailurops | ursinus | 19.35 | 35.51 | 3.25 | B | 3 | 10.00 |
| M | Spilocuscus | maculatus | 18.52 | 34.30 | 3.16 | B | 1 | 6.40 |
| M | Trichosurus | caninus | 13.77 | 18.50 | 3.20 | B | 1 | 3.50 |
| M | Trichosurus | vulpecula | 16.81 | 21.38 | 3.63 | B | 1 | 2.65 |
| M | Phascolarctus | cinerus | 25.22 | 54.98 | 3.37 | B | 5 | 10.25 |
| M | Aepyprymnus | rufescens | 12.19 | 21.06 | 2.66 | B | 1 | 3.25 |
| M | Potorous | tridactylus | 12.62 | 15.73 | 3.18 | I | 2 | 1.10 |
| M | Hemibelideus | lemuroides | 16.21 | 12.60 | 4.54 | B | 2 | 0.95 |
| M | Petauroides | volans | 13.14 | 11.43 | 3.87 | B | 2 | 1.30 |
| M | Pseudocheirops | archeri | 18.26 | 19.53 | 4.13 | B | 1 | 1.09 |
| M | Pseudochirops | cupreus | 16.34 | 23.37 | 3.42 | B | 2 | 1.72 |
| M | Thylacinus | cynocephalus | 15.19 | 77.23 | 1.73 | C | 1 | 30.00 |
| M | Lasiorhinus | latifrons | 26.25 | 82.23 | 2.89 | G | 1 | 25.50 |
| M | Vombatus | ursinus | 23.35 | 78.74 | 2.65 | G | 14 | 26.00 |
| E | Atelerix | albiventris | 9.27 | 14.26 | 2.46 | I | 1 | 0.60 |
| E | Erinaceus | concolor | 12.98 | 17.47 | 3.10 | I | 1 | 0.72 |
| E | Hemiechinus | auritus | 8.86 | 20.34 | 1.97 | I | 1 | 0.34 |
| E | Equus | asinus | 78.51 | 608.96 | 3.18 | G | 1 | 165.00 |
| E | Equus | caballus | 90.36 | 658.99 | 3.52 | G | 1 | 250.00 |
| E | Equus | grevyi | 101.22 | 738.73 | 3.72 | G | 1 | 408.00 |
| E | Equus | hemionus | 102.54 | 667.94 | 3.43 | G | 1 | 230.00 |
| E | Ceratotherium | simum | 183.31 | 2904.38 | 3.40 | G | 1 | 2949.99 |
| E | Diceros | bicornis | 136.16 | 2285.07 | 2.85 | B | 2 | 1180.51 |
| E | Tapirus | bairdii | 31.71 | 398.62 | 1.59 | H | 1 | 299.99 |
| E | Tapirus | indicus | 58.56 | 602.01 | 2.39 | H | 2 | 296.25 |
| E | Aotus | lemurinus | 10.82 | 9.29 | 3.55 | H | 4 | 0.87 |
| E | Alouatta | seniculus | 25.54 | 57.74 | 3.36 | B | 3 | 6.15 |
| E | Colobus | guerza | 27.33 | 38.96 | 4.40 | B | 3 | 10.20 |
| E | Nasalis | larvatus | 32.70 | 54.97 | 4.41 | B | 2 | 9.00 |
| E | Theropithecus | gelada | 28.20 | 69.36 | 3.38 | G | 2 | 17.05 |
| E | Pan | troglodytes | 20.04 | 100.28 | 2.01 | H | 3 | 45.00 |
| E | Avahi | laniger | 12.42 | 17.13 | 3.00 | B | 1 | 0.90 |
| E | Lemur | catta | 15.51 | 28.21 | 2.92 | B | 1 | 2.90 |
| E | Lepilemur | mustelinus | 14.31 | 15.28 | 3.66 | B | 1 | 0.77 |
| E | Cacajao | melanocephalus | 7.63 | 14.83 | 1.98 | H | 2 | 3.80 |
| E | Callicebus | torquatus | 7.88 | 5.52 | 3.29 | H | 2 | 1.28 |
| E | Chiropotes | satanas | 10.92 | 16.65 | 2.68 | H | 1 | 3.00 |
| E | Pithecia | pithecia | 7.80 | 14.65 | 2.04 | H | 2 | 1.38 |
| E | Microtus | pennsylvanicus | 11.24 | 7.57 | 4.08 | H | 1 | 0.04 |
| E | Handleyomys | alfaroi | 13.19 | 55.67 | 1.77 | I | 1 | 0.03 |
| E | Nyctomys | sumichrasti | 4.93 | 2.10 | 3.38 | H | 2 | 0.06 |
| E | Ototylomys | phyllotis | 6.40 | 3.85 | 3.27 | H | 3 | 0.12 |
| E | Peromyscus | leucopus | 2.62 | 1.25 | 2.36 | H | 2 | 0.02 |
| E | Tylomys | nudicaudus | 6.10 | 4.27 | 2.95 | H | 3 | 0.10 |
| E | Mus | bufo | 1.87 | 0.85 | 1.99 | H | 2 | 0.01 |
| E | Oenomys | hypoxanthus | 6.79 | 4.53 | 3.19 | H | 3 | 0.09 |
| E | Rattus | rattus | 3.53 | 1.73 | 2.68 | H | 1 | 0.28 |
| E | Tatera | leucogaster | 3.19 | 3.74 | 1.66 | I | 5 | 0.07 |
| E | Oxymycterus | paramensis | 3.53 | 2.21 | 2.06 | H | 1 | 0.04 |
| E | Peromyscus | maniculatus | 57.24 | 331.79 | 3.14 | H | 1 | 0.02 |
| E | Zygodontomys | brevicaudata | 3.33 | 1.95 | 2.38 | H | 1 | 0.05 |
| E | Steatomys | pratensis | 1.95 | 1.89 | 1.42 | H | 2 | 0.03 |
| E | Aeromys | thomasi | 11.01 | 11.50 | 3.24 | H | 3 | 1.44 |
| E | Callosciurus | nigrovittatus | 8.94 | 4.76 | 4.09 | H | 3 | 0.20 |
| E | Callosciurus | notatus | 8.83 | 5.33 | 3.80 | H | 4 | 0.19 |
| E | Callosciurus | prevostii | 6.93 | 5.01 | 3.14 | H | 3 | 0.40 |
| E | Hylopetes | lepidus | 5.66 | 2.02 | 3.98 | H | 1 | 0.12 |
| E | Hylopetes | spadiceus | 5.57 | 2.04 | 3.90 | H | 3 | 0.05 |
| E | Iomys | horsfieldi | 7.51 | 4.24 | 3.65 | H | 5 | 0.12 |
| E | Lariscus | insignis | 3.91 | 4.53 | 1.84 | H | 2 | 0.20 |
| E | Petaurista | elegans | 10.75 | 9.74 | 3.45 | H | 1 | 0.95 |
| E | Ratufa | affinis | 10.60 | 9.70 | 3.40 | H | 3 | 1.13 |
| E | Ratufa | bicolor | 9.75 | 12.24 | 2.79 | H | 3 | 2.05 |
| E | Sciurus | alleni | 9.54 | 6.98 | 3.61 | H | 1 | 0.43 |
| E | Sciurus | carolinensis | 8.46 | 6.11 | 3.41 | H | 2 | 0.51 |
| E | Sundasciurus | hippurus | 9.65 | 5.83 | 4.00 | H | 1 | 0.08 |
| E | Sundasciurus | tenuis | 3.68 | 2.33 | 2.41 | H | 1 | 0.50 |
| E | Solenodon | paradoxus | 13.40 | 25.48 | 2.65 | I | 5 | 0.90 |
| E | Blarina | brevicauda | 7.40 | 3.29 | 4.08 | I | 2 | 0.03 |
| E | Chimarrogale | himalayica | 9.01 | 4.95 | 4.05 | I | 1 | 0.04 |
| E | Nectogale | elegans | 7.47 | 3.23 | 4.17 | I | 3 | 0.03 |
| E | Neomys | fodiens | 6.29 | 2.71 | 3.82 | I | 4 | 0.14 |
| E | Scutisorex | somereni | 8.87 | 7.07 | 3.36 | I | 3 | 0.09 |
| E | Sorex | araneus | 4.13 | 1.40 | 3.49 | I | 2 | 0.01 |
| E | Soriculus | nigrescens | 6.54 | 2.95 | 3.78 | I | 3 | 0.01 |
| E | Suncus | murinus | 11.29 | 7.78 | 4.06 | I | 2 | 0.07 |
| E | Condylura | cristata | 5.99 | 3.21 | 3.34 | I | 2 | 0.08 |
| E | Neurotrichus | gibbsei | 4.97 | 2.47 | 3.16 | I | 1 | 0.01 |
| E | Parascalops | breweri | 6.18 | 4.15 | 3.07 | I | 3 | 0.05 |
| E | Scalopus | aquaticus | 7.32 | 5.19 | 3.21 | I | 5 | 0.09 |
| E | Talpa | europaea | 6.42 | 3.66 | 3.36 | I | 2 | 0.08 |

**SECTION 3**

The methodology used in this study was adapted from Solounias and Semprebon 2002 [28]. Molds of museum specimens were made on-site, then casts were made for use in the analysis. Molding was done using standard methods: vinylpolysloxane molding compound (3M ESPE) regular set, light body was extruded onto the tooth after first removing surface debris with an ethanol swab. Casts were made using clear Buehler Epo-Kwik epoxy resin.

*1. Orientation of the specimen and light source*

Using modeling clay, the tooth cast is oriented so that the tooth facet to be analyzed is horizontal. Lighting is provided using a single light of a gooseneck lamp sitting to one side of the microscope. Microwear analysis was performed at 70x magnification by counting the number of features in a square standardized reticle 0.2 mm on a side.

Measurements are taken under two different lighting conditions: one (referred to here as Bright Field or BF) so that the background appears bright with the wear features showing up dark, and the other (Dark Field or DF) having a dark background with wear features showing up bright. This provides a qualitative measure of feature size, with the larger features showing up dark on a light background (tallied in the categories *Lp* and *Cs)* and smaller features showing up bright on a dark background (tallied in the categories *Sp* and *Fs*). The number of features counted in both bright and dark fields are then tallied into the total scratch (*Ts*) and total pit (*Tp*) categories. Figure 1, below, illustrates the angle of the gooseneck light required to produce BF and DF. BF images are produced at an angle of approximately 100° +/- 5° and DF at 140° +/- 5°.

*Figure S1*. Schematic depicting the orientation of the specimen under the reflected-light microscope. Pictures are of the field of view under changing unidirectional light from the right side of the image; the number next to each image refers to the angle of the light source.





**SECTION 4**

Because microwear is derived from physical contact with the food source it is inherently more variable than tooth morphology (here represented by SCS) or body mass, both of which have genetic components. In order to evaluate the degree to which increased sample size might influence the global data set, the limited data set (comprised only of the species represented by three or more individuals, up to the maximum of eight) (Table S3, below) was subjected to the same statistical tests as the global data set, yielding similar results. After the data were log-transformed, nested ANOVAs performed on the limited data set reveal the same three statistically significant clusters as the global data set: Browsers, Grazers, and Hard-Object Feeders (Grazers:browsers p<0.0001, browsers:hard-object p<0.0001, grazers:hard-object p<0.0001). Because no eutherian browser is represented by three or more individual animals, the metatherian/eutherian comparison could not be made for the total pit and scratch counts. Post-hoc results for individual variable pairings are remarkable similar to those obtained from analysis of the global data set (Table S4, see Table 1 for comparison): While the p-values may differ between the two data sets, in only four cases (marked with * in Table S4) did that result in a change in significance for that pairing. Importantly, all of the total pit and total scratch pairings yield the same significance results in both data sets.

Discriminate analysis tests of the three herbivorous guilds using the four independent microwear variables *Fs, Cs, Sp*, and *Lp* resulted in well-defined clusters and a post-hoc leave-one-out correct assignment rate remarkably similar to that of the global data set: of 84.2% (global data set 82.8%) for the three herbivore guilds, the same test including all five guilds had a correct assignment rate of 57.1% (global data set 57.2%).

| **Diet Code** | **Genus** | **Species** | **# Individuals** |
| --- | --- | --- | --- |
| 1 | Crocuta | crocuta | 3 |
| 1 | Felis | sylvestris | 5 |
| 1 | Hyaena | hyaena | 4 |
| 1 | Panthera | leo | 3 |
| 1 | Panthera | pardus | 3 |
| 2 | Callosciurus | prevostii | 3 |
| 2 | Ototylomys | phyllotis | 3 |
| 2 | Ratufa | bicolor | 4 |
| 2 | Tapirus | bairdii | 3 |
| 3 | Dendrolagus | lumholtzi | 4 |
| 3 | Hemibelideus | lemuroides | 3 |
| 3 | Petaruoides | volans | 6 |
| 3 | Phascolarctos | cinereus | 4 |
| 3 | Setonix | brachyurus | 5 |
| 3 | Spilocuscus | maculatus | 4 |
| 3 | Trichosurus | vulpecula | 3 |
| 4 | Equus | caballus | 3 |
| 4 | Macropus | fuliginosus | 3 |
| 4 | Macropus | giganteus | 4 |
| 4 | Macropus | robustus | 8 |
| 4 | Petrogale | lateralis | 3 |
| 5 | Gerbilliscis | leucogaster | 3 |
| 5 | Hemigalus | derbyanus | 3 |
| 5 | Netogale | elegans | 3 |
| 5 | Neomys | fodiens | 3 |
| 5 | Perameles | nasuta | 4 |
| 5 | Potorous | tridactylus | 3 |

*Table S3*. Taxon list of species included in the limited data set, with the number of individuals representing each species. Diet Category: I=Insectivore, C=Carnivore, G=Grazer, B=Browser, H=Hard-Object Feeder.

*Table S4*: Statistical results of univariate ANOVA tests on the limited data set, including post-hoc LSD test between individual pairings. Significant p-values for the LSD post-hoc tests are in bold, showing the dietary guilds with significant differences for each microwear variable. Pairings with an LSD significance result different than that obtained for that pairing in the global data set are marked with an asterisk (*).

|  |  |  |  |  | **LSD Post-Hoc Results: p-values** | | |  |  |  |  |  |  |  |
| --- | --- | --- | --- | --- | --- | --- | --- | --- | --- | --- | --- | --- | --- | --- |
|  | **Variable** | ***F*** | ***d.f.*** | ***p*** | C-HO | C-B | C-G | C-I | HO-B | HO-G | HO-I | B-G | B-I | G-I |
| Microwear: Scratches | *S_f_* | 6.53 | 4 | <0.001 | **0.008** | 0.331 | **<0.001** | **0.012** | **0.039** | 0.224 | 0.673 | **<0.001** | *0.066 | 0.068 |
|  | *S_c_* | 19.34 | 4 | <0.001 | **0.017** | **<0.001** | *0.077 | 0.084 | **<0.001** | 0.425 | **0.023** | **<0.001** | **<0.001** | 0.099 |
|  | *S_t_* | 15.12 | 4 | <0.001 | **<0.001** | 0.498 | **<0.001** | **0.001** | **<0.001** | 0.379 | 0.423 | **<0.001** | **<0.001** | 0.084 |
| Microwear: Pits | *P_s_* | 26.28 | 4 | <0.001 | **0.008** | **<0.001** | **<0.001** | **0.002** | **<0.001** | **<0.001** | **0.002** | 0.063 | **<0.001** | **<0.001** |
|  | *P_l_* | 4.08 | 4 | 0.004 | **0.017** | 0.192 | 0.521 | 0.556 | **<0.001** | **0.003** | *0.058 | 0.514 | **0.049** | *0.208 |
|  | *P_t_* | 22.43 | 4 | <0.001 | **0.002** | **<0.001** | **<0.001** | 0.889 | **<0.001** | **<0.001** | **0.002** | 0.093 | **<0.001** | **0.001** |
| Body Mass | *BM* | 49.35 | 4 | <0.001 | **<0.001** | 0.156 | 0.662 | **<0.001** | **<0.001** | **<0.001** | **0.037** | **0.005** | **<0.001** | **<0.001** |
| Shearing Crest Score | *SCS* | 13.39 | 4 | <0.001 | **<0.001** | **<0.001** | **<0.001** | **<0.001** | 0.063 | 0.495 | 0.894 | **0.016** | **0.045** | 0.572 |

*Table S5***.** Results of independent-samples T-tests for bone carnivores vs. flesh carnivores.

No significant differences were detected.

|  | *Variable* | p |
| --- | --- | --- |
| Microwear: Scratches | *Fs* | 0.939 |
|  | *Cs* | 0.088 |
|  | *Ts* | 0.631 |
| Microwear: Pits | *Sp* | 0.060 |
|  | *Lp* | 0.525 |
|  | *Tp* | 0.778 |
| Body Mass | *BM* | 0.105 |
| Shearing Crest Score | *SCS* | 0.205 |

**SCS Values and Body Size**

**A**

**B**


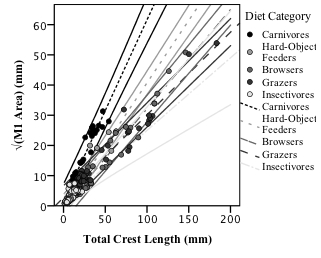

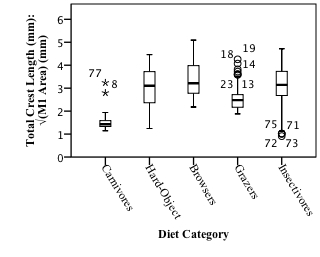


*Figure S2*. Shearing crest length relative to tooth size for different dietary guilds. A) The absolute shearing length of an animal’s molar has little discriminative value by itself, but is effective in distinguishing diets among animals of similar size. Carnivores have the steepest slope, shearing crest length increasing the least with respect to molar size, allowing this dietary habit to be identified by tooth morphology alone (no other trajectory within 95% confidence). Grazers/browsers, by contrast, increase crest length to a greater degree as they get bigger for more efficient breakdown of tough plant mater. Grazers and browsers are indistinguishable here, which is not unexpected, but together are outside the 95% confidence intervals for both carnivores and hard-object feeders (and can be reliably differentiated from each other based on microwear, as discussed in the main text). Hard-object feeders have more crest development than carnivores of similar size but less than high-fiber herbivores of comparable size. The insectivores fall into a small cloud for which there is no clear relationship between tooth size and crest length over the limited range they occupy (R^2^ = 0.12). **B)** Body mass distributions of the species included in analysis, separated by feeding guild. Insectivores occupy the smallest end of the body mass spectrum, as do hard-object feeders with rare exceptions (tapirs and some primates). Large insectivores such as anteaters and aardwolves are also exceptions due to the fact that they feed on colonial insects; because they have very simple and reduced numbers of teeth, they were excluded from this study. Browsers have a minimum body size of about 500g (members of the genus *Lepilemur*).
